# A Review of the Efficacy and Safety for Biologic Agents Targeting IL-23 in Treating Psoriasis With the Focus on Tildrakizumab

**DOI:** 10.3389/fmed.2021.702776

**Published:** 2021-08-10

**Authors:** Feras M. Ghazawi, Farhan Mahmood, Leon Kircik, Yves Poulin, Marc Bourcier, Ronald Vender, Marni C. Wiseman, Charles Lynde, Ivan V. Litvinov

**Affiliations:** ^1^Division of Dermatology, University of Ottawa, Ottawa, ON, Canada; ^2^Faculty of Medicine, University of Ottawa, Ottawa, ON, Canada; ^3^Department of Dermatology, Mount Sinai Hospital, New York City, NY, United States; ^4^Division of Dermatology, Laval University, Quebec City, QC, Canada; ^5^Faculty of Medicine, University of Sherbrooke, Sherbrooke, QC, Canada; ^6^Division of Dermatology, McMaster University, Hamilton, ON, Canada; ^7^Section of Dermatology, Department of Medicine, University of Manitoba, Winnipeg, MB, Canada; ^8^Division of Dermatology, University of Toronto, Toronto, ON, Canada; ^9^Division of Dermatology, McGill University, Montréal, QC, Canada

**Keywords:** psoriais, treatment, tildrakizumab, guselkumab, IL-23, risankizumab, mirikizumab, ustekinumab

## Abstract

Psoriasis is a chronic and debilitating inflammatory immune-mediated skin disorder. Several cytokines including interleukin (IL)-23 were demonstrated to play a central role in the pathogenesis of this disease. Treatment options for psoriasis range from topical to systemic modalities, depending on the extent, anatomical locations involved and functional impairment level. Targeting cytokines or their cognate receptors that are involved in disease pathogenesis such as IL-12/23 (i.e., targeting the IL-12p40 subunit shared by these cytokines), IL-17A, IL-17F, IL-17RA, and TNF-α using biologic agents emerged in recent years as a highly effective therapeutic option for patients with moderate-to-severe disease. This review provides an overview of the important role of IL-23 signaling in the pathogenesis of psoriasis. We describe in detail the available IL-23 inhibitors for chronic plaque psoriasis. The efficacy, pharmacokinetic properties, and the safety profile of one of the most recent IL-23 biologic agents (tildrakizumab) are evaluated and reviewed in depth.

## Introduction

**Figure 1:**
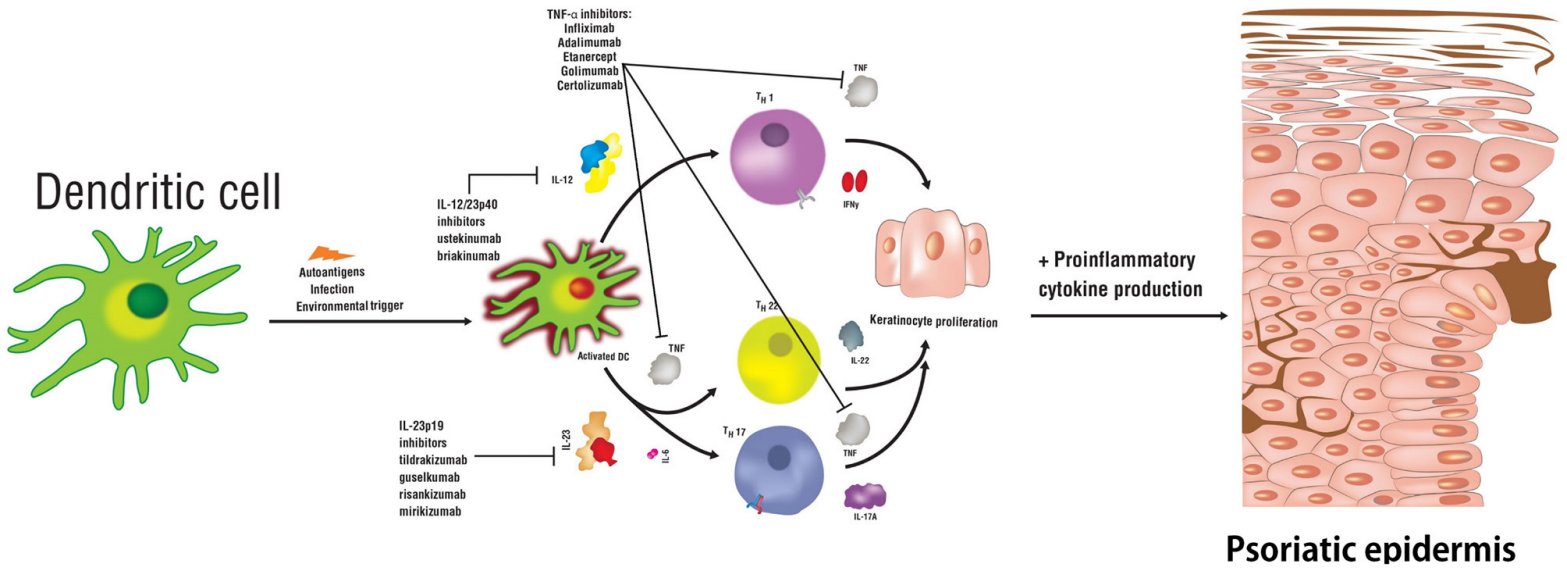
Schematic representation of relevant cytokines and T helper cell subsets involved in the pathogenesis of psoriasis. Biologic therapies used to treat this disease are indicated along with the signaling pathways targeting specific cytokines.

Psoriasis is a T-cell mediated autoimmune inflammatory disease that primarily affects the skin but can also affect the joints and other organs. The prevalence of psoriasis in the developed countries is between ~1–5% (1), with the most common clinical form being the chronic plaque subtype. The underlying etiology of psoriasis is multifactorial and is comprised of genetic predisposition, immunologic, environmental, and endogenous factors (2). These factors ultimately affect various components of innate and adaptive immunity to result in dysregulated keratinocyte proliferation and the development of psoriatic lesions. Psoriasis is a heterogenous disease, where >80 genes and alleles were described to increase disease susceptibility, including *HLA-Cw6, PSOR1-15, CCHCR1, CDSN* along with the range of inflammatory molecules regulated by the TNF-α signaling pathway in T helper (Th) cells (Figure 1) (3, 4).

## The Role of Interleukin-23 Signaling in the Pathogenesis of Psoriasis

**Figure 2:**
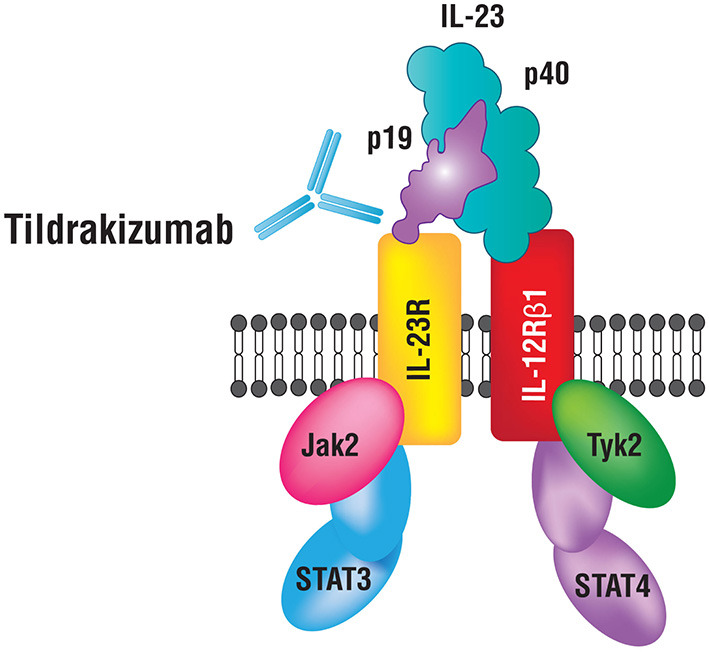
IL-23 and its receptor complex. IL-23 is a heterodimeric cytokine composed of p40 and p19 subunits.

IL-23 has been shown to play a fundamental role in the pathogenesis of psoriasis (2). IL-23 is a heterodimeric cytokine composed of two subunits p19 and p40 (Figure 2). The p19 subunit is unique to the structure of IL-23, a 4-fold helical core with a disulfide bond, which is attached to the p40 subunit (5, 6). The p40 subunit is shared with IL-12, where it dimerizes with the p35 subunit (7). Genomic studies have confirmed that IL-23p19 is found on chromosome 12q13.2; the gene is composed of four exons and three introns, whereas, IL-23p40 is located on chromosome 11q1.3, composed of eight exons and seven introns (5–8). Antigen-presenting cells (APCs) including Langerhans cells, macrophages, and tissue-resident or recruited inflammatory myeloid CD11^+^ dendritic cells (DC) produce IL-23. Keratinocytes were also shown to produce mRNA transcripts for IL-23p19 and IL-23p40 (9). Various immune factors are involved in the expression of IL-23 by APCs including lipopolysaccharides, CpG, and PolyI:C (10). These factors bind to toll-like receptors (TLRs) to induce the activation of transcription factors AP-1 and NF-kB, which then further leads to the upregulation of IL-23 (11).

A key immunologic function of IL-23 is to drive the differentiation process of naïve T-helper (Th) cells into Th17 cells, primary producers of IL-17. Studies demonstrate that the presence of IL-6, IL-1β, or TGF-β is not sufficient for Th17 differentiation and that concomitant IL-23 stimulation is essential. IL-23 inhibits the differentiation of regulatory T (Treg) cells that produce IL-10 and inhibit inflammation, and thus restrict Th17 differentiation (12, 13). IL-6, IL-1 β, and TGF-β are essential for the expression of IL-17A, IL-17F, and IL-23 receptors (IL-23R) (6, 14). IL-23 binds to a receptor complex to induce biological inflammatory responses (Figure 2). The receptor complex is comprised of two parts, IL-12Rβ1 and IL-23R and is expressed by several immune cells including natural killer, dendritic and memory T-cells, macrophages, and keratinocytes (14–16). Upon binding to the receptor complex, specifically on naïve Th cells, IL-23 activates Signal Transducer and Activator of Transcription (STAT3), which then dimerizes, translocates into the nucleus and binds/transactivates the promoters of IL-17A and IL-17F (17). IL-17 is an essential cytokine involved in linking T-cell activation to neutrophil mobilization and activation of the Th17 inflammatory pathway in several autoimmune conditions including psoriasis (7, 8, 18). IL-23 and IL-12 together activate Th17 and Th1 cells that release IL-22 and TNF-α (19). IL-22 is an effector cytokine of the Th17 lineage and works cooperatively with IL-17A and IL-17F (20). Furthermore, IL-23 plays a role in promoting differentiation of CD8^+^ T cells into cytotoxic T 17 cells (Tc17). Tc17 cells along with mast cells, neutrophils, and IL-23R^+^ T cells further increase IL-17 production upon stimulation by IL-23 (21–24). Intriguingly, recent data from studies in mice indicates that tissue-resident innate lymphoid 3 cells (ILC3) also produce IL-17 and IL-22 cytokines in response to IL-23 signaling, which contributes to dermal inflammation in psoriasis (25).

Thus, IL-23 stimulation and the expression of downstream cytokines secreted by T cells, CD4^+^-T cells, regulatory T cells, cytotoxic T cells, natural killer cells, type 3 innate lymphoid cells, neutrophils, and mast cells are observed in psoriasis (26). T-cell activation inhibitors are known to significantly ameliorate psoriatic lesions, albeit, none are used for the treatment of disease due to significant adverse effects (27–30). Deregulation of genes related to the IL-23/Th17 signaling axis increases the risk of developing psoriasis. Psoriatic skin lesions have consistently demonstrated increased levels of IL-23 (specifically IL-23p19 and p40 mRNAs), IL-17, IL-22, and infiltration of epidermal Th17 cells as well as dermal Tc17 cells (22, 31–33). IL-23 protein levels were also higher in psoriatic skin, when compared to non-lesional skin (34). Intradermal injection of IL-23 in murine skin models led to histological changes consistent with psoriatic lesions. Consistent with this finding, experimental imiquimod-induced psoriasis models are dependent on IL-23 and IL-17 production (35–37). Blocking of the IL-23/IL-17 signaling axis using anti-IL-23 antibodies was shown to suppress the onset of psoriasis in experimental animals (38). IL-23p19 and IL-17RA deficient mice showed an amelioration in erythema, scaling, and skin thickening (37). Injections of recombinant IL-23 into mice stimulated epidermal hyperplasia and psoriasis plaques formation through IL-17 and IL-22 signaling, which was not observed in IL-17 and IL-22 deficient mice (39). IL-22 is known to directly induce keratinocyte proliferation and migration (40). IL-17A increases proliferation of keratinocytes and downregulates the expression of molecules involved in their differentiation. IL-17A and IL-22 interact with TNF-α to upregulate the expression of IL-36, a cytokine that further augments the function of Th17 cytokines creating a feedback loop observed in pustular psoriasis (41).

As detailed above, IL-23 and IL-12 share the same p40 subunit that binds to their receptor complexes to initiate an immune response. IL-12 binds to CD4^+^ T cells via the IL-12 receptor complex, which triggers the differentiation of naïve T cells into Th1 cells. Th1 cells release IFN-γ and TNF-α. Together with IL-23, IL-12 increases the production of pro-inflammatory cytokines involved in the pathogenesis of psoriasis (19).

In summary, in the pathogenesis of psoriasis, IL-6, IL-1β, and TGF-β initiate the differentiation of naïve Th17 cells, alongside IL-23, which is required for Th17 activation and maintenance, and secretion of pro-inflammatory cytokines (Figure 1). IL-23 is crucial for the survival and proliferation of Th17 cells, primary producers of IL-17. IL-17 protein is observed at high levels in blood and skin samples from psoriasis patients. IL-23 signaling also leads to the release of IL-22, which together with IL-17, further stimulates keratinocytes to produce chemokines and antimicrobial peptides to recruit additional Th17 cells, therefore, sustaining the inflammatory response (42, 43). Concomitantly, IL-12 induces the differentiation of the Th1 cells and triggers the release of IFN-γ and TNF-α (7, 19, 22, 39, 41, 44–47).

## Inhibition of the Il-12P40 Subunit Shared by the Il-12/23 Cytokines for the Treatment of Psoriasis

The key role of IL-23 in the pathogenesis of psoriasis makes this cytokine an intriguing therapeutic target (48–52). Clinical studies have shown that inhibition of IL-23 effectively treats symptoms of psoriasis, as several other key inflammatory cytokines including IL-17, IL-22, TNF-α, and IL-36 are inhibited (2). Although, the focus of this review is on IL-23 inhibition and particularly on tildrakizumab (53–55), we briefly discuss inhibitors targeting TNF-α, IL-17/IL-17RA, and IL-12/23. TNF-α inhibitors include etanercept, infliximab, adalimumab, certolizumab pegol, and golimumab (the latter used off-label to treat psoriasis) (56); IL-17 inhibitors include secukinumab, ixekizumab, and bimekizumab; IL-17RA inhibitor–brodalumab (19, 57–61); Inhibitors of IL-12p40 subunit, which affect the IL-12 and IL-23 signaling include ustekinumab and briakinumab. The status of regulatory approval by Health Canada, the U.S. Food and Drug Administration (FDA), and the European Medicine Agency (EMA) for these drugs is summarized in Table 1.

**Table 1:** Summary of status of approval for the treatment of psoriasis by Health Canada, the Food and Drug Administration (FDA), and the European Medicines Agency (EMA) of biologic agents presented in this review.

**Biologic agent**	**Approved by health Canada (62)**	**Approved by the Food and Drug Administration (63)**	**Approved by the European Medicine Agency (64)**
Ustekinumab	Yes	Yes	Yes
Briakinumab	No	No	No
Guselkumab	Yes	Yes	Yes
Risankizumab	Yes	Yes	Yes
Mirikizumab	Trials ongoing	Trials ongoing	Trials ongoing
Tildrakizumab	Under review	Yes	Under review
Etanercept	Yes	Yes	Yes
Infliximab	Yes	Yes	Yes
Adalimumab	Yes	Yes	Yes
Certolizumab pegol	Yes	Yes	Yes
Golimumab (for psoriatic arthritis only)	Yes	Yes	Yes
Secukinumab	Yes	Yes	Yes
Ixekizumab	Yes	Yes	Yes
Bimekizumab	Under review	Under review	Under review
Brodalumab	Yes	Yes	Yes

### Ustekinumab

Discovery of elevated expression of the p40 subunit in psoriatic lesions combined with biologic plausibility prompted the development of targeted biologic therapies. The first agent was an entirely humanized antibody, ustekinumab (48). Ustekinumab targets IL-23 and IL-12 by neutralizing IL-23p40 to treat chronic plaque psoriasis and psoriatic arthritis. Ustekinumab was approved by the FDA at the dose of 45 or 90 mg as an injection depending on the patient's weight. A randomized controlled clinical trial demonstrated that ustekinumab demonstrated superior efficacy than etanercept in treating psoriasis over a 12-week period (50). Two large randomized controlled clinical trials with the total of 1,996 patients with moderate-to-severe psoriasis demonstrated that ustekinumab 45 and 90 mg achieved a 75% reduction in the psoriasis area and severity index (PASI; PASI75) more significantly than the placebo (51, 52). Further clinical trials have assessed the long-term safety and efficacy of ustekinumab and have produced similar results (50, 65–67). Specifically, one long-term study illustrated that 76.5 and 78.6% of patients were demonstrating a PASI75 response after a 5-year period of ustekinumab 45 and 90 mg treatment, respectively (65). The adverse events (AEs) of this drug were studied across a 3-year period demonstrating comparable findings between the placebo vs. 45 and 90 mg doses of ustekinumab (50).

### Briakinumab

Briakinumab is another entirely humanized antibody that was developed to inhibit p40 subunit, however drug development was discontinued (2). Initially, clinical trials suggested that this agent was effective and safe (68, 69). A randomized controlled study, where 347 chronic plaque psoriasis patients were given briakinumab, etanercept, or placebo, showed that briakinumab was superior to etanercept (68). Another randomized controlled, double-blind clinical trial demonstrated that briakinumab was more effective than methotrexate. However, concern about increased occurrence of serious AEs including infections, malignancies, and, importantly, cardiac events led to the discontinuation of briakinumab's development (40).

## Targeted Inhibition of IL-23 Signaling as a Reliable Systemic Treatment Strategy for Psoriasis

Therapeutic agents specifically targeting IL-23p19 subunit include guselkumab, tildrakizumab, risankizumab, and mirikizumab.

### Guselkumab

Guselkumab is a humanized IgG1 lambda monoclonal antibody used to treat moderate-to-severe chronic plaque psoriasis (70). The dosage is 100 mg administered at weeks 0, 4 and then every 8 weeks thereafter (71–73). An initial randomized controlled trial with 24 participants at week 12 demonstrated a PASI75 response in guselkumab treated patients at a significantly higher rate than in patients receiving a placebo (73). Two randomized controlled trials (VOYAGE 1 and VOYAGE 2) compared the efficacy and safety of guselkumab 100 mg, adalimumab (a TNF-α inhibitor), a commonly used biologic at that time for chronic plaque psoriasis, and a placebo. In VOYAGE 1 trial, 73.3% of patients using guselkumab achieved a PASI90 disease response at week 16, when compared to 49.7% of patients using adalimumab. VOYAGE 2 trial demonstrated a PASI90 response of 70 and 2% for guselkumab vs. placebo groups, respectively. Investigator Global Assessment (IGA 0/1) improvement was also significantly greater for the guselkumab compared to the adalimumab and placebo groups, with 85 vs. 65.9% and 7% of patients achieving IGA 0/1, respectively, at week 16. Both studies showcased the long-term efficacy of guselkumab up to 48 weeks. AEs were comparable across all groups (72, 74). Another randomized controlled trial included patients receiving ustekinumab 45 or 90 mg at weeks 0 and 4. At week 16, patients with inadequate responses to ustekinumab (defined as maintaining an IGA score of ≥2) were re-randomized to receive guselkumab 100 mg or they continued the same ustekinumab treatment. This trial illustrated that at week 28, 48.1% of patients after switching to guselkumab achieved a PASI90 response rate in comparison to 22.6% of patients continuing to receive ustekinumab. IGA improvements for the guselkumab vs. ustekinumab arms of the study were observed in 31 and 14% of the patients, respectively, at week 28. Thus, guselkumab was shown to be a superior alternative for ustekinumab in patients, who do not respond to IL-12/23 p40 inhibitor. However, 66.4% of patients receiving guselkumab had an AE compared to 55.6% treated with ustekinumab; the most frequent being common non-severe infections (75).

The systematic review and Bucher indirect comparison of tildrakizumab and guselkumab demonstrated that one treatment is not superior to the other, according to the results from the reSURFACE 1/2, and VOYAGE 1/2 trials. There were no statistically significant differences between the two biologic agents in achieving PASI75 and 90 scores or serious AEs (76). The rates of discontinuation at weeks 12 to 16 and 24 to 28 were comparable between the drugs (76). The data for the outcomes of the placebo groups for weeks 24 to 28 was imputed from weeks 12 to 16 due to the discontinuation of the placebo arm after week 16. The authors assumed that changes are not expected in the placebo arm beyond weeks 12 to 16, which is a limitation of this study design.

### Risankizumab

Risankizumab is a human monoclonal antibody of IgG1 class that also targets the IL-23p19 subunit for the treatment of psoriasis (a dosing schedule of 150 mg at weeks 0, 4, and subsequently every 12 weeks) (77). A phase III randomized controlled trial compared the efficacy of risankizumab with adalimumab in patients with moderate-to-severe chronic plaque psoriasis (78). In total, 605 patients were enrolled in this study and randomized to receive risankizumab or adalimumab. Seventy two percent of patients in the risankizumab group achieved a PASI90 score compared to 47% of patients in the adalimumab group at week 16. Subsequently, 66% of patients in the adalimumab group were able to achieve a PASI90 score at week 44, after switching to receive risankizumab treatment, while only 21% of patients, who continued the treatment with adalimumab, achieved a PASI90 score. AEs were similar across all groups. Two phase III multicenter trials (ultIMMa-1 and ultIMMa-2) were further conducted to compare risankizumab 150 mg *vs*. placebo *vs*. ustekinumab (79). Both trials demonstrated risankizumab to be more effective than the placebo and ustekinumab; ultIMMa-1 trial illustrated that 75.3% of patients achieved a PASI90 score at week 16 using risankizumab compared to 4.9 and 42% of patients in the placebo and ustekinumab groups, respectively. At week 52, 82, and 56% of patients receiving risankizumab achieved PASI90 and PASI100 scores in the ultIMMa-1 trial, similar to 81 and 60%, respectively in the ultIMMa-2 trial. Treatment-emergent AEs were consistent across all groups, most of which included the upper respiratory tract infections (URTIs), fatigue, headache, injection-site reaction, and dermatophyte infections. A 2-year trial further assessed the efficacy and safety of continuous use of risankizumab. Participants receiving risankizumab achieved a PASI90 clearance at a significantly higher rate than the placebo group: 73.2 vs. 2% of patients, respectively, at week 16. The rates of AEs remained stable and were comparable to those observed in the placebo arm over the 2 years (80). An immunohistochemical analysis of 81 psoriasis patients treated with risankizumab for 4 weeks showed a significant decrease in immunohistochemical marker staining associated with psoriasis including K16, Ki67, CD3, and CD11c in 69% of patients receiving 180 mg dosing. Similar molecular changes were observed in only 29% of patients treated with ustekinumab (81).

### Mirikizumab

More recently, mirikizumab, a humanized monoclonal IgG4-variant antibody, was developed as an IL-23 antagonist. This biologic agent is currently being studied for its potential use in psoriasis and Crohn's disease/Ulcerative colitis patients. A multicentre phase II randomized controlled trial assessed the efficacy and safety of mirikizumab in treating moderate-to-severe chronic plaque psoriasis. In total, 205 patients were randomized into either a placebo *vs*. mirikizumab 30 mg, 100 mg, or 300 mg groups, where injections were administered at weeks 0, 8, and then every 8 weeks thereafter. At week 16, 67 and 59% of patients in the mirikizumab 100 and 300 mg groups, respectively, achieved a PASI90 score, compared to 0% in the placebo group. AEs were similar across all groups. Hypertension was observed in 5 patients receiving mirikizumab 100 mg and 300 mg groups, along with viral infections/URTIs, injection-site pain and diarrhea, and were similar across all dosage groups. Serious AEs included suicidal tendencies, observed once in both placebo and mirikizumab groups, and alanine aminotransferase and aspartate aminotransferase enzyme elevation >10 times of the upper limit of normal, observed once in the mirikizumab group. Further studies are required to demonstrate that this biologic agent is an effective and safe therapeutic option for psoriasis (82).

### Tildrakizumab

Tildrakizumab is a high affinity humanized monoclonal IgG kappa IL-23p19 antibody (83–94). Tildrakizumab (SCH-900222, MK03222) was developed by Merck, Sun Pharmaceutical Industries and approved by the FDA in March 2018 for the treatment of moderate-to-severe chronic plaque psoriasis in adults (95–97). Tildrakizumab was the second IL-23p19 inhibitor approved by the FDA after guselkumab (98). Tildrakizumab binds to IL-23p19 and inhibits its interaction with the IL-23 receptor. The recommended dose is 100 mg administered on 0, 4 weeks, and then every 12 weeks thereafter. However, it is up to the physician's discretion to escalate the dose to 200 mg, when necessary (99). Tildrakizumab is recommended as the first line monotherapy for moderate-to-severe psoriasis (100). This antibody is available in 1 mL syringes at 100 mg/ml concentrations. The pre-filled syringes should be stored in a refrigerator and left at room temperature for 30 min before use.

In 2015, a phase 1, randomized placebo-controlled trial evaluated the efficacy of tildrakizumab in treating chronic plaque psoriasis. This initial trial has demonstrated PASI75 score in all subjects treated with intravenous tildrakizumab 3 and 10 mg/kg by day 196 in two out of the three parts of this phase 1 trial (101). These successful results were followed by a phase 2b trial assessing the safety and efficacy of subcutaneous tildrakizumab in moderate-to-severe chronic plaque psoriasis (102). Tildrakizumab's efficacy and safety was superior to placebo, maintaining response for 52 weeks of treatment and persisting for 20 weeks after cessation. However, this trial was limited due a small sample size, requiring a larger phase 3 trial assessing the safety and tolerability of tildrakizumab. In 2017, the results from two phase 3 trials were published. These studies illustrated that tildrakizumab 100 and 200 mg doses are more effective and well-tolerated compared to the placebo and etanercept in treating moderate-to-severe chronic plaque psoriasis (103). However, these trials were limited because comparisons with more effective TNF-α inhibitors or ustekinumab have not been conducted. The non-responders treated with tildrakizumab discontinued therapy before part 3, which started at week 28 of the trial. This resulted in lower dropout rates in these treatment arms within 28 weeks. The authors also noted that 12 weeks may have been too early to assess the efficacy of tildrakizumab adequately. Thus, in-between-treatment analyses for tildrakizumab 100 mg were not conducted at several endpoints, including PASI75 and PGA responses at 28 weeks. Currently, four other trials are ongoing to assess the efficacy and safety of tildrakizumab. These include the extension phase 3 reSURFACE 1 and reSURFACE 2, two multinational, phase 2 trials, a multiple-dose phase 2b study in patients with active psoriatic arthritis, and a phase 2a study in patients with active ankylosing spondylitis or non-radiographic axial spondylarthritis. The extensions of reSURFACE 1 and 2 are observational studies designed to further assess the efficacy profile of tildrakizumab and its adverse events (103, 104).

## Efficacy of Tildrakizumab in the Treatment of Psoriasis: Detailed Review of Clinical Trial Data

As highlighted above, a phase I, randomized controlled trial with 77 subjects demonstrated a PASI75 response in participants treated with the 3 and 10 mg/kg intravenous tildrakizumab after 196 days from the first dose. This study also included a histological, immunohistochemical, and gene expression analyses of psoriatic skin following the treatments. Individuals treated with 3 mg/kg and 10 mg/kg of intravenous tildrakizumab experienced a resolution of thickened psoriatic skin lesions and demonstrated reduced epidermal hyperplasia as well as a decrease of vascular and inflammatory cell infiltrate parameters. All of the groups had a significant reduction in the histopathological psoriasis severity score, a mean reduction of 67%. Proliferation markers including Ki67 and keratin 16, apparent in psoriasis, also normalized upon treatment, along with the inflammatory infiltrating cells (epidermal CD4^+^ and CD8^+^ T-cells, dermal myeloid DCs, plasmacytoid DCs, and CD15^+^ neutrophils). Tildrakizumab treatment reduced and normalized the levels of IL-19, IL-20, CCL20 ligands (CCL20 is overexpressed in psoriasis and binds to Th17 chemokine receptors), and CXCL8/IL-8 (overexpressed in psoriasis and binds to neutrophil CXCE1/2 receptors) in the lesional skin (101).

A phase II randomized controlled trial with 355 patients affected by chronic plaque psoriasis demonstrated that subcutaneous injections of tildrakizumab resulted in PASI75 clearance that was maintained through 1 year. The most potent response was produced using the tildrakizumab 200 mg treatment, where PASI75 clearance was achieved in 74.4% of patients at week 16, compared to 66.3% in patients receiving 100 mg dosing and 4.4% in the placebo group (102). Two randomized controlled phase III trials (reSURFACE 1 and reSURFACE 2) were conducted to compare the efficacy and safety of tildrakizumab (*n* = 1,863 subjects). In part 1 of the reSURFACE 1 trial, participants received tildrakizumab 100, 200 mg, or placebo treatments subcutaneously at 0 and 4 weeks. In part 2, tildrakizumab treated patients received doses at week 16, and the placebo group patients were re-randomized to receive either tildrakizumab 100 or 200 mg for weeks 12 and 16 doses. In the reSURFACE 2 trial, during part 1 participants received tildrakizumab 100, 200 mg, a placebo treatment, or etanercept 50 mg (50 mg twice a week for 12 weeks then 50 mg once weekly for 16 weeks). In part 2 of this trial, tildrakizumab group patients received doses at week 16, while the re-randomized placebo group patients received tildrakizumab 100 or 200 mg for weeks 12 and 16 doses. In part 3 of both trials, responders and partial responders (PASI ≥ 75 and PASI ≥50 or PASI <75, respectively) to tildrakizumab treatment were re-randomized to continue the same regimen, an alternative tildrakizumab dosing, or a placebo treatment for subsequent doses. Patients with missing data were treated as non-responders and in these cases data imputation was carried our. However, for secondary analyses, the full-analysis-set observed data (i.e., all randomized participants who received one or more doses of treatment and had baseline and one or more post-baseline efficacy measurements) was conducted.

The results demonstrated at week 12, that tildrakizumab 100 and 200 mg treatments were significantly more effective than the placebo and etanercept groups in achieving PASI75 clearance. In the reSURFACE 2 trial, 61% and 66% of patients receiving tildrakizumab 100 mg or 200 mg, respectively, achieved a PASI75 response score compared to 48, and 6% in etanercept and placebo groups, respectively. AEs were similar and occurred at low frequency across all groups (103).

Combined data from phase IIb and III trials demonstrated that PASI75 was achieved using tildrakizumab 200 mg (62–74%), 100 mg (61–64%), 25 mg (59%), 5 mg (24%) doses, in comparison to a placebo and etanercept: 4–6 and 48%, respectively at week 12. The phase III reSURFACE trials also demonstrated that ~70% of tildrakizumab patients achieved a Physician Global Assessment (PGA) score of clear or almost clear. A review of phase II and III trial data using tildrakizumab established that the most common AEs included common, non-threatening infections.

Recently the data became available highlighting 5-year efficacy and safety outcome based on long-term extension of the reSURFACE 1 and 2 trails (105). The results highlighted that patients who responded to tildrakizumab (i.e., achieved PASI 75 at week 28) 100 mg treatment demonstrated PASI 75/90/100 response at week 244 at the rate of 88.7, 65.9, and 32.8%, respectively. For the 200 mg treatment, responders at week 28 continued to demonstrate clinical benefit at week 244 as 92.5% of patients achieved PASI75, while 69.5 and 40.8% of patients achieved PASI90 and PASI100 responses, respectively. Subjects that demonstrated a partial response to etanercept and non-responders were switched to receive tildrakizumab 200 mg treatment. These patients benefitted and achieved PASI75/90/100 and at rates of 81.3, 49.5, and 21.5%, respectively. Five-year analysis of safety data was comparable to findings of shorter time studies, where the most frequent treatment related AE was nasopharyngitis. Several severe AEs were observed in both tildrakizumab 100 mg and 200 mg groups and were deemed not related to treatment (105). Hence, long-term continuous dosing or switching from another biologic agent could be part of the psoriasis management regimen using tildrakizumab.

Furthermore, the impact of patient demographic and disease characteristics on tildrakizumab efficacy was studied. PASI75 and 90 scores were achieved slightly more frequently (not reaching statistical differences) in patients, who were <65 years of age, and had a bodyweight of <90 kg, no evidence of arthritis, and no prior biologic exposure. The efficacy of tildrakizumab did not differ based on sex, race, and prior failure of conventional systemic treatments (106). To further assess the efficacy of tildrakizumab 100 mg treatment on scalp, head and neck psoriasis disease at week 28 a *post-hoc* analysis was conducted using the data from the reSURFACE 1 trial. A PASI head component score (PASIh) (range 0.0–7.2) was used. Rapid, progressive reduction in PASIh score was noted at week 28. Tildrakizumab's efficacy for scalp, head and neck clearance has shown to be similar to secukinumab and adalimumab treatments, however, there are no direct comparison studies available (107). A *post-hoc* analysis also demonstrated that tildrakizumab treated patients with PASI > 75 at week-28 maintained their improvement at week 52, and >50% of the partial responders at week 28 improved their PASI scores to more than 75 at week 52 (108). Gordon et al. further evaluated supplementing dichotomous efficacy with residual disease activity and found that disease activity was more reliably estimated by PASI scores than PASI improvements by percentages. At week 12, the median PASI score was 2.9 and the response rate for PASI 90 was 36.9%, whereas, at week 28, the median PASI was 1.7 and the response rate for PASI 90 was 51.9% (109). Another *post-hoc* analysis assessed the time and predictors leading to relapse in patients treated with tildrakizumab 100 and 200 mg. The median time to loss of PASI 75 from 28 weeks was 142 and 172 days with 100 and 200 mg dosing, respectively. Increase in body mass index and an increase in disease duration were associated with relapse (110).

## Cost Effectiveness

Tildrakizumab has been shown to be cost-effective. The introduction of tildrakizumab with a 1% annual uptake over 5 years can potentially reduce the cost of treating psoriasis patients based on the data from the United States. In a population of 1,048 psoriasis patients treated with tildrakizumab, the total health plan costs decreased by $964,763 over the span of 5 years. Tildrakizumab is one of the most cost-effective first-line psoriasis treatments. It has been shown to be more cost-effective than risankizumab, secukinumab, guselkumab, ixekizumab, adalimumab, ustekinumab, etanercept, and certolizumab pegol (111–113).

## Pharmacokinetic Properties of Tildrakizumab

The bioavailability of tildrakizumab is ~73–80% following an injection (96). The half-life of tildrakizumab is ~20.2–28.2 days, with the low volume of distribution of ~10.8 L. Intravenous administration of tildrakizumab 0.1, 0.5, 3, or 10 mg/kg produced mean half-life times of 29.4, 29.7, 26.9, and 24.6 days, respectively (104). Dosing with 100 mg of tildrakizumab on weeks 0, 4, and every 12 weeks thereafter resulted in a steady state achieved by week 16, where the mean steady-state concentration ranged from 1.22 ± 0.94 to 1.47 ± 1.12 mcg/mL (99). Tildrakizumab is most likely cleared via catabolic pathways that degrade this immunoglobulin into small peptides and amino acids. However, the pharmacokinetic properties of tildrakizumab and its use in geriatric, pediatric, breastfeeding, or pregnant female populations have not been extensively studied (96, 99). Other pharmacokinetic parameters including maximum concentration and area under the curve of tildrakizumab increase proportionally from doses of 50–200 mg. Increased body weight resulted in lower area under the plasma concentration-time curve at steady state (114). The pharmacokinetic properties of tildrakizumab are similar to other monoclonal antibodies and do not require dosage adjustments.

To better understand IL-23 pharmacokinetic parameters, the degree of target suppression associated with clinical efficacy, accelerator mass spectrometry was used to measure the concentration of human recombinant [^14^C]-IL-23 in cynomolgus monkeys. It was found that the predicted rank order of reduction of free IL-23 was consistent with the reported rank order of PASI100 scores in clinical efficacy trials; the rankings were ustekinumab < tildrakizumab < guselkumab < risankizumab (115).

It was also found that the pharmacokinetic factors such as half-life, maximum concentration, drug exposure over time, and median time to drug concentration were comparable across different races and/or ethnicities including Chinese, Japanese and Caucasian. The overall geometric mean area under the receiver operating curve was 6.15, 6.05, and 6.32-day × μg/mL/mg for Japanese, Caucasian and Chinese subjects, respectively, upon treatment with tildrakizumab (116). Across all three populations, 10 mg/kg dosing was well-tolerated. Other studies demonstrated that 50 mg of tildrakizumab dosing had a bioavailability of 80% and 200 mg dosing had a bioavailability of 73% (101, 116–118).

The effect of tildrakizumab on cytochrome P450 metabolism was studied in psoriasis patients, as changes in systemic inflammation have been shown to alter cytochrome P450 metabolism. Tildrakizumab showed no clinically relevant effects on the pharmacokinetic properties of the probe substrates. There were also no changes in the IL-6 or high-sensitivity C-reactive protein levels (119).

A few studies have assessed the immunogenicity of tildrakizumab evaluating antidrug antibody (ADA) production. Between 8 and 18% of patients tested positive for an ADA after a treatment with tildrakizumab. Two of the studies demonstrated decreased levels of tildrakizumab in patients with positive ADAs, and another demonstrated a decrease in tildrakizumab half-life in ~30% of subjects with ADAs. However, further studies are required to understand the immunogenicity of tildrakizumab and the relevance of antibody suppressants in ADA positive patients (102, 116, 118). Finally, another study evaluating ADA development in psoriasis patients established a dose-dependent relationship, where 6.5 and 8.2% of treatment-emergent ADAs occurred using tildrakizumab 100 mg and 200 mg dosing regimens, respectively. Specifically, the incidence of treatment-emergent ADA-positive *neutralizing* antibodies was 2.5 and 3.2% for tildrakizumab 100 and 200 mg, respectively. In patients with a positive ADA, when compared to a negative ADA status, the efficacy of tildrakizumab decreased. At week 52, the mean PASI score improvement in treatment-emergent neutralizing antibody-positive vs. ADA negative patients were 76% (*n* = 10) vs. 91% (*n* = 342) for the 100 mg of tildrakizumab dosing regimen. Thus, participants with treatment-emergent ADAs and neutralizing antibodies showed reduced efficacy and lower serum levels of tildrakizumab (120).

## Safety Profile of Tildrakizumab

As highlighted by the data from clinical trials, tildrakizumab is a reliable treatment leading to only minor AEs, including an increased risk of nasopharyngitis/URTIs (99). To test safety, 29 healthy subjects were randomized to receive either 0.1, 0.5, 3, or 10 mg/kg doses of tildrakizumab, or a placebo treatment intravenously. Of the subjects receiving intravenous tildrakizumab, 83% reported at least one AE, 50% in 0.1 mg/kg; 100% in 0.5 mg/kg; 88% in 3 mg/kg; 100% in 10 mg/kg; and 71% in the placebo groups. The most common AEs were URTIs, headaches, injection-site reactions, and fatigue. Serious AEs included upper airway obstruction observed in the placebo group, and rhinal surgery observed in the 10 mg/kg treatment group (117). In a different study, 37 healthy subjects were randomized to receive 50 or 200 mg of tildrakizumab, or placebo subcutaneously. Subcutaneous injections resulted in AEs in 65% of participants; 64% in 50 mg; 57% in 200 mg; and 78% in the placebo group. Common AEs included URTIs and headaches, and no serious AEs were observed (117). Also, while the product monograph advises to evaluate patients for a possible latent or active tuberculosis infection, as per the typical “biologic classification,” because of the targeted nature of this treatment, the risk of reactivating or increasing susceptibility to tuberculosis remains low (121).

The incidence of serious gastrointestinal disorders, including inflammatory bowel disease (Crohn's disease and ulcerative colitis), were assessed in detail using the data from phase IIb and III trials (118, 122, 123). The *post-hoc* analysis based on 28 weeks of clinical trial data concluded that tildrakizumab did not induce or worsen inflammatory bowel disease in patients with psoriasis. This is in contrast to the clinical trials using IL-17 and IL-17RA inhibitors that demonstrated new cases and exacerbation of inflammatory bowel disease in psoriasis and Crohn's disease patients (124).

Furthermore, as mentioned above the long-term safety and tolerability of tildrakizumab has been evaluated using the data from tildrakizumab clinical trials for up to 244 weeks (105). Consistent with our review, frequencies of treatment-emergent AEs, serious AEs, discontinuations due to AEs, major adverse cardiovascular events, and severe infections were comparable between tildrakizumab 100 mg, tildrakizumab 200 mg, placebo, and etanercept groups in the reSURFACE 1 and 2 trials. There were no AEs of inflammatory bowel disease or suicides reported up to 244 weeks of tildrakizumab use. Candida skin infections were infrequent with rates of 0.1, 0.3, 0.0, and 0.0% for the tildrakizumab 100 mg, tildrakizumab 200 mg, placebo and etanercept groups, respectively (105, 125).

Psoriasis is associated with the metabolic syndrome and portends a higher risk of cardiovascular disease (126). A *post-hoc* analysis of the tildrakizumab trials demonstrated that the efficacy, safety, and drug survival were comparable and similar in psoriasis patients with or without a metabolic syndrome (127). A recent study demonstrated that the cardiometabolic risk factors for patients receiving tildrakizumab 100 mg or 200 mg doses differed at week 52 and 64 compared to the baseline. To evaluate cardiometabolic risk, fasting serum glucose, low/high-density lipoprotein-cholesterol, total cholesterol, triglyceride levels, body weight, and blood pressure were studied (128). It was confirmed that patients with psoriasis treated with tildrakizumab 100 or 200 mg doses up to 3 years were not susceptible to increased cardiovascular risk (129).

Another important consideration is the use of tildrakizumab during pregnancy. A recent consensus paper regarding the management of chronic plaque psoriasis with biologic therapies in women of child-bearing potential stated that tildrakizumab could be transported across the placenta and there is a possibility that it can be present in breast milk. A *post-hoc* analysis of clinical trials analyzed 14 women who received tildrakizumab and became pregnant. The outcomes included 6 cases of fetal loss (2 spontaneous and 4 elective abortions) and 8 live births. There were no congenital anomalies observed. This study does not demonstrate a clear association, as it is unknown whether tildrakizumab led to the spontaneous abortions in the aforementioned 2 cases. It is worth noting that ~10–15% of all natural pregnancies end in recognized spontaneous abortions (130). Hence, 2/14 spontaneous abortion rate is comparable to that in the general population. Nevertheless, female patients should use contraceptive measures, when treated with tildrakizumab and should refrain from using this biologic agent if pregnant until more data becomes available (131). To our knowledge, there are no studies conducted evaluating the safety of tildrakizumab during breast feeding. Due to tildrakizumab's large molecular structure, it is unlikely to be absorbed by the infant and is likely to be metabolized by the infant's gastrointestinal tract (132, 133).

The safety profile of tildrakizumab was also assessed using a cynomolgus monkey model. Cynomolgus monkeys were treated with 100 mg/kg of tildrakizumab every 2 weeks up to 9 months and the drug was found to be well-tolerated at systemic exposures approximately 90 times higher than what is recommended for human patients. Treatment with tildrakizumab 100 mg/kg in pregnant monkeys did not lead to embryofetal developmental abnormalities (134).

Tildrakizumab is contraindicated in patients with a previous serious hypersensitivity to this drug or to any of the excipients. If a hypersensitivity reaction occurs, use should be discontinued. The use of live vaccines should also be restricted. Prior to initiating tildrakizumab, age-appropriate immunizations should be completed according to the immunization guidelines (99).

## Comparing Efficacy and Safety Profiles of Tildrakizumab

There are no direct head-to-head comparison clinical studies evaluating the efficacy and safety of tildrakizumab to other biologic agents aside from etanercept, as presented earlier. In Tables 2–4 and Table 1 in Appendix, we compared the efficacy/safety of tildrakizumab in treating psoriasis with clinical trials conducted using other biologic agents: IL-23 inhibitors [i.e., guselkumab (72, 74), risankizumab (79), mirikizumab (82)], IL-23/12p40 inhibitor [i.e., ustekinumab (51, 52)], IL-17/IL-17 receptor inhibitors [i.e., secukinumab (135), ixekizumab (123), brodalumab (136), and bimekizumab (137)], and TNF-α inhibitors [i.e., etanercept, infliximab (138), adalimumab (139), certolizumab pegol (140), and golimumab (141)]. We indicated PASI75, 90, and 100 scores, PGA measures, common adverse and serious AEs from each pivotal clinical trial and compared demographic parameters of the study population.

**Table 2:** Summary of the demographic data of patients enrolled in clinical trials testing the efficacy of IL-23, IL-23/12, IL-17, IL-17RA, and TNF-α inhibitors.

**References**	**Phase**	**Biologic**	**Dosing scheme**	**Endpoint week**	**Total (*n*)**	**Age (years)**	**Male (%)**	**Weight (kg)**	**BMI (kg/m ∧ 2)**	**Disease duration (years)**	**% body surface**
Resurface 1 (103) (dosing scheme summary only from part 1)	3	Tildrakizumab	100 mg, week 0, 4	12	309	46.4	67	88.5			29.7
		Tildrakizumab	200 mg, week 0, 4	12	308	46.9	73	88.9			30.9
		Placebo		12	154	47.9	65	87.5			29.6
Resurface 2 (103) (dosing scheme summary only from part 1)	3	Tildrakizumab	100 mg, week 0, 4	12	307	44.6	72	89.4			34.2
		Tildrakizumab	200 mg, week 0, 4	12	314	44.6	72	88.4			31.8
		Etanercept	50 mg, week 0, 4	12	313	45.8	71	88			31.6
		Placebo		12	156	46.4	72	88.7			31.3
Papp et al. (102)	2b	Tildrakizumab	5 mg, week 0, 4	12	42	43.2	74		28.9		
		Tildrakizumab	25 mg week 0, 4	12	92	46.3	65		28.5		
		Tildrakizumab	100 mg week 0.4	12	89	45.5	85		29		
		Tildrakizumab	200 mg week 0, 4	12	86	43.2	76		28.5		
		Placebo		12	46	45.9	83		29.5		
Kopp et al. (101)	1	Tildrakizumab	3 mg/kg–weeks 0, 4	28	7	52.7	100	90.27			
		Tildrakizumab	10 mg/kg–weeks 0, 4	28	6	46.2	67	94.25			
		Placebo		28	20	45.5	80	102.46			
Voyage 1 (72)	3	Guselkumab	100 mg–week 0, 4	16	329	43.9	72.9		29.7	17.9	28.3
		Placebo		16	174	44.9	68.4		28.9	17.6	25.8
Voyage 2 (74)	3	Guselkumab	100 mg–week 0, 4	16	496	43.7	70.4		29.6	17.9	28.5
		Placebo		16	248	43.3	69.8		29.6	17.9	28
UltIMMa-1 (79)	3	Risankizumab	150 mg–week 0, 4	16	304	48.3	70	87.8			26.2
		Placebo		16	102	49.3	77	88.8			27.9
UltIMMa-2 (79)	3	Risankizumab	150 mg–week 0, 4	16	294	46.2	69	92.2			26.2
		Placebo		16	98	46.3	68	92.2			23.9
Reich et al. (82)	2	Mirikizumab	100 mg–week 0, 8	16	51	46	69	86.4		18.6	26.5
		Mirikizumab	300 mg–week 0, 8	16	51	47.5	71	87.9		18.1	21.3
		Placebo		16	52	46	81	89.1		18	26.4
Phoenix-1 (51)	3	Ustekinumab	90 mg–week 0, 4	12	256	46.2	67.6	93.8		19.6	25.2
		Placebo		12	255	44.8	71.8	94.2		20.4	27.7
Phoenix-2 (52)	3	Ustekinumab	90 mg–week 0, 4	12	411	46.6	66.7	91.5		20.3	27.1
		Placebo		12	410	47	69	91.1		20.8	26.1
FIXTURE (135)	3	Secukinumab	150 mg–weeks 0–4 then every 4 weeks thereafter	12	327	45.4	72.2	83.6		17.3	34.5
		Secukinumab	300 mg–weeks 0–4 then every 4 weeks thereafter	12	327	44.5	68.5	83		15.8	34.3
		Placebo		12	326	44.1	72.7	82		16.6	35.2
		Etanercept	50 mg–weeks 0–4 then every 4 weeks thereafter	12	326	43.8	71.2	84.6		16.4	33.6
UNCOVER-1 (123)	3	Ixekizumab	160 mg × 1, 80 mg (q2w)	12	433	45	67.2	92		20	27
		Ixekizumab	160 mg × 1, 80 mg (q4w)	12	432	46	66.9	92		19	28
		Placebo		12	431	46	70.3	92		20	27
AMAGINE-1 (136)	3	Brodalumab	140 mg q2w	12	219	46	74	90.6		19	27.4
		Brodalumab	210 mg q2w	12	222	46	73	91.4		20	25.1
		Placebo		12	220	47	73	90.4		21	26.9
BE ABLE 1 (137)	2b	Bimekizumab	160 mg q4w	12	40	43.4	65.1	91.6		15.9	24
		Bimekizumab	320 mg q4w	12	43	42.6	74.4	86.9		15.9	24
		Placebo		12	42	46.7	59.5	88.8		15	25.5
EXPRESS II (138)		Infliximab	5 mg/kg	14	314	44.5	65	92.2		19.1	28.7
		Placebo		14	208	44.4	69.2	91.1		17.8	28.4
REVEAL (139)	3	Adalimumab	40 mg q2w	15	814	44.1	67.1	92.3		18.1	25.8
		Placebo		15	398	45.4	64.6	94.1		18.4	25.6
CIMPACT (140)	3	Certolizumab pegol	200 mg q2w	16	165	46.7	68.5	89.7		19.5	28.1
		Placebo		16	57	46.5	59.6	93.7		18.9	24.3
GO-VIBRANT (141)	3	Golimumab	2 mg/kg, weeks 0, 4 then every 8 weeks thereafter	16	241	45.7	53.1			6.2	
		Placebo		16	239	46.7	50.6			5.3	

**Table 3:** Summary of AEs and serious AEs that occurred in clinical trials evaluating the efficacy and safety of IL-23, IL-23/12, IL-17, and TNF-α inhibitors.

**References**	**Biologic**	**Dose**	**Sample Size**	**Week #**	**>/= 1 AEs reported %**	**Common AE types listed**	**>/= 1 SAEs reported %**	**Common SAE types listed**
Resurface 1 (103)	Tildrakizumab	100 mg	309	12	47	8%Nasopharyngitis; 3% URTIs; 1% psoriasis	2	<1% severe infection; <1% confirmed major adverse cardiovascular events
		200 mg	308	12	42	2% discontinued use; 6% nasopharyngitis; 5% URTIs	3	<1% severe infection; <1% Drug-related hypersensitivity
	Placebo	n/a	154	12	48	1% discontinued use; 5% nasopharyngitis; 6% URTIs; 5% psoriasis	1	
Resurface 2 (103)	Tildrakizumab	100 mg	307	12	44	1% discontinued use; 1% injection site erythema; 13% nasopharyngitis	1	<1% death; <1% malignancies; <1% non-melanoma skin cancer; <1% drug-related hypersensitivity
		200 mg	314	12	49	1% discontinued use; 1% injection site erythema; 11% nasopharyngitis	2	<1% severe infections <1% malignancies; <1% non-melanoma skin cancer
	Etanercept	50 mg	313	12	54	2% discontinued use; 1% injection site erythema; 8% nasopharyngitis	2	1% severe infections; 1% drug-related hypersensitivity
	Placebo		156	12	55	1% discontinued use; 1% injection site erythema; 11% nasopharyngitis	3	1% severe infections; 1% drug-related hypersensitivity
Papp et al. (102)	Tildrakizumab	5 mg	42	16	71	2% discontinued use	0	
	Tildrakizumab	25 mg	92	16	61	2% discontinued use; 1% injection site reaction	1	1% bacterial arthritis
	Tildrakizumab	100 mg	89	16	65	1% discontinued use; 1% serious infections	1	1% death
	Tildrakizumab	200 mg	86	16	63	1% discontinued use	2	1% ovarian cyst; 1% lymphoedema
	Placebo		46	16	69	1% discontinued use; 1% injection site reaction	0	
Kopp et al. (101)	Tildrakizumab	3 mg/kg	6	16	71	14% headache; 14% cough; 14% nasopharyngitis; 14% arthralgia; 14% back pain; 14% hypertension		
	Tildrakizumab	10 mg/kg	5	16	33			
	Placebo		20	16	75	15% headache; 15% cough; 10% nasopharyngitis; 15% arthralgia; 5% back pain; 5% hypertension; 15% URTI; 5% oropharyngeal pain; 10% fatigue; 5% pruritis; 15% sinusitis; 10% psoriasis		Only 1 serious AE (convulsions) was deemed possibly related to tildrakizumab
Voyage 1 (72)	Guselkumab	100 mg	329	16	51.70	9.1% nasopharyngitis; 7.6% URTIs; 1.8% injection-site erythema; 3.6% headache; 3.3% arthralgia; 1.5% pruritis; 1.8% back pain	2.40	0.3% NMSC; 0.3% MACE
	Placebo		174	16	49.40	9.8% nasopharyngitis; 5.2% URTIs; 4% headache; 1.7% arthralgia; 5.7% pruritis; 1.1% back pain	1.70	
Voyage 2 (74)	Guselkumab	100 mg	496	16	47.60	7.1% nasopharyngitis; 5.1% headache; 3.2% URTIs; 21.5% infections	1.60	0.2% serious infections
	Placebo		248	16	44.80	6.5% nasopharyngitis; 2.8% headache; 4% URTIs; 18.5% infections	1.20	0.4% serious infections
UltIMMA-1 (79)	Risankizumab	150 mg	304	16	49.7	24.7% infections;	4.3	0.3% serious infection; 0.3% malignancies
	Placebo		102	16	51	16.7% infections	7.8%	1% malignancies
UltIMMA-2 (79)	Risankizumab	150 mg	294	16	45.6	19% infections	4.40	1% severe infections; 0.3% malignancies; o.3% deaths
	Placebo		98	16	45.9	9.2% infections	2	
Reich et al. (82)	Mirikizumab	100 mg	51	16	47	25% infections; 20% URTIs; 4% injection site pain; 6% hypertension 2% diarrhea	0	
	Mirikizumab	300 mg	51	16	47	25% infections; 9% URTIs; 4% injection-site pain; 4% hypertension; 6% diarrhea	2	
	Placebo		52	16	48	23% infections; 7% URTIs; 2% injection-site pain; 2% diarrhea	2	
Phoenix-1 (51)	Ustekinumab	90 mg	256	12	51.4	6.3% URTIs; 8.2% nasopharyngitis; 2.4% arthralgia; 5.1% headache	1.6	25.9% infections; 0.8% serious infections
	Placebo		255	12	48.2	6.3% URTIs; 8.6% nasopharyngitis; 2.7% arthralgia; 2.4% headache	0.8	26.7% infections; 0.4% serious infections
Phoenix-2 (52)	Ustekinumab	90 mg	411	12	47.9	2.4% arthralgia; 1% cough; 4.6% headache; 1.5% injection-site erythema; 3.4% URTI; 6.8% nasopharyngitis	1.20%	22.4% infection; 0.2% serious infection; 0.2% skin cancer; 0.2% CV event
	Placebo		410	12	49.8	2.9% arthralgia; 1.7% cough; 4.1% headache; 0.2% injection-site erythema; 7.1% nasopharyngitis; 3.4% URTIs	2%	20% infections; 0.5% serious infections; 0.2% cutaneous cancer; 0.2% non-cutaneous cancer
FIXTURE (135)	Secukinumab	150 mg	327	12	58.4	13.8% NP; 4.9% Headache; 3.7% diarrhea; 3.7% pruritis; 4.3% arthralgia; 3.1% URTI; 2.4% back pain; 1.5% cough; 3.1% hypertension; 1.8% nausea; 1.5% oropharyngeal pain	2.10%	30.9% infections
	Secukinumab	300 mg	323	12	55.5	10.7% NP; 9.2% Headache; 5.2% diarrhea; 2.5% pruritis; 1.5% arthralgia; 2.1% URTI; 2.5% back pain; 3.4% cough; 1.5% hypertension; 2.5% nausea; 2.8% oropharyngeal pain	1.20%	26.7% infections
	Etanercept	50 mg	323	12	57.6	11.1% NP; 7.1% Headache; 3.4% diarrhea; 2.5% pruritis; 3.7% arthralgia; 2.2% URTI; 2.8% back pain; 1.2% cough; 1.5% hypertension; 1.2% nausea; 1.2% oropharyngeal pain	0.90%	24.5% infections
	Placebo		324	12	49.8	8% NP; 7% Headache; 1.8% diarrhea; 3.4% pruritis; 3.1% arthralgia; 0.9% URTI; 1.8% back pain; 1.2% cough; 1.2% hypertension; 2.1% nausea; 2.1% oropharyngeal pain	1.80%	19.3% infections
UNCOVER-1 to 3 (123)	Ixekizumab	160 mg × 1, 80 mg × q2w	1,167	12	58.4	9.5% nasopharyngitis;4.4% URTI; 10% injection site reaction; 2.5% Arthralgia; 4.4% headache	1.7	27% infections; 0.1% cancer; 0.2% nonmelanoma skin cancer; 0.1% Chron's disease
	Ixekizumab	160 mg × 1, 80 mg × q4w	1,161	12	58.8	9% nasopharyngitis; 3.9% URTI; 7.7% injection site reaction; 1.9% arthralgia; 4.3% headache	2.2	27.4% infections; 0.2% Mace; 0.1% Crohn's disease; 0.2% cancer; 0.1% non-Melanoma skin cancer
	Placebo		791	12	46.78	8.7% nasopharyngitis; 3.5% URT; one point 1% injection site reaction; 2.1% arthralgia; 2.9% headache	1.5	22.9% infections; 0.1% Mace; 0.1% cancer; 0.1% non-Melanoma skin cancer
AMAGINE-1 (136)	Brodalumab	140 q2w	219	12	57.5	0.5% depression; 1.4% injection site reaction; 0.5% neutropenia; 9.1% nasopharyngitis; 8.2% URTI; 5.5% headache	2.7	0.9% serious infectious episode
	Brodalumab	210 q2w	222	12	59	0.5% depression; 0.5% injection site reaction; 9.5% nasopharyngitis; 8.1% URTI; 5% headache	1.8	0.5% serious infectious episode
	Placebo		220	12	50.9	0.5% depression; 10% nasopharyngitis; 6.4% URTI; 3.2% headache	1.4	
BE ABLE 1 (137)	Bimekizumab	160 mg q4w	40	12	55.8	7% nasopharyngitis; 4.7% URTI; 7% glutamyl transferase increase; 2.3% hypertension 2.3%; respiratory tract infection; 4.7% tonsillitis; 2.3% rhinitis	0	
	Bimekizumab	320 mg q4w	43	12	60.5	14% nasopharyngitis; 4.7% URTI; 2.3% arthralgia; 2.3% glutamyl transferase increase; 2.3% respiratory tract infection; 4.7% neutropenia	0	
	Placebo		42	12	35.7	4.8% nasopharyngitis; 2.4% URTI; 2.4% glutamyl transferase increase; 2.4% rhinitis; 7.1% hypertension; 2.4% respiratory tract infection	2.4	
EXPRESS II (138)	Infliximab	5 mg/kg	314	14	68.8	13.4% URTI; 12.1% headache; 5.1% pharyngitis; 3.8% nausea; 4.5% pain; 6.4% sinusitis; 2.9% pruritis; 1.9% coughing; 2.9% rhinitis; 2.2% hypertension; 1.6% psoriasis	2.9	30.9% infections
	Placebo		208	14	56	14% URTI; 5.3% headache; 3.4% pharyngitis; 3.9% nausea; 4.3% pain; 1.4% sinusitis; 4.3% Pruritis; 1.4% coughing; 0.5% rhinitis; 3.9% hypertension; 4.8% psoriasis	2.4	30% infections
REVEAL (139)	Adalimumab	40 mg	814	15	62.2	28.9% infections; 7.2% URTI; 5.3% nasopharyngitis; 4.9% headache	1.8	0.6% serious infection; 0.2% malignancies; 0.5% non-melanoma skin cancer;
	Placebo		398	15	55.5	22.4 infections; 3.5% URTI; 6.5% nasopharyngitis; 3.8% headache	1.8	1% serious infection; 0,3% malignancy; 0.3% non-melanoma skin cancer
CIMPACT (140)	Certolizumab pegol	200 mg	165	12	47.3	8.5% nasopharyngitis; 3.6% URTI; 0.6% depression	0.6	26.7% infection and infestations
	Placebo		57	12	56.1	8.8% nasopharyngitis; 10.5% URTI	8.8	28.1% infection and infestations
GO-VIBRANT (141)	Golimumab	2 mg/kg	241	24	46.3	0.4% demyelinating events; 0.8% injection site reaction	2.9	45% infections; 0.4% serious infections
	Placebo		239	24	40.6		3.3	0.8% serious infections; 0.8% malignancies; 0.8% deaths; 15.5% infections

**Table 4:** Summary of PASI75, 90, and 100 responses evaluating biologic agents in Phase III clinical trials for IL-23, IL-23/12, IL-17, and TNF-α inhibitors.

**Biologic**	**Dose**	**PASI 75**		**PASI 90**		**PAS 100**	
Tildrakizumab (103)	100 mg	64% at 12 wks	80% at 28 wks	35% at 12 wks	52% at 28 wks	14% at 12 wks	24% at 28 wks
	200 mg	62% at 12 wks	82% at 28 wks	35% at 12 wks	59% at 28 wks	14% at 12 wks	32% at 28 wks
Tildrakizumab (103)	100 mg	61% at 12 wks	73% at 28 wks	39% at 12 wks	56% at 28 wks	12% at 12 wks	23% at 28 wks
	200 mg	66% at 12 wks	73% at 28 wks	37% at 12 wks	58% at 28 wks	12% at 12 wks	27% at 28 wks
Guselkumab (72)	100 mg	91.2% at 16 wks		73.3% at 16 wks		37.4% at 16 wks	
Guselkumab (74)	100 mg	86.3% at 16 wks		70% at 16 wks		34.1% at 16 wks	
Risanzikumab (79)	150 mg	86.8% at 12 wks		75.3% at 16 wks		35.9% at 16 wks	
Risanzikumab (79)	150 mg	88.8% at 12 wks		74.8% at 16 wks		50.7% at 16 wks	
Mirikizumab (82)	100 mg	78% at 16 wks		59% at 16 wks		31% at 16 wks	
Mirikizumab (82)	300 mg	75% at 16 wks		67% at 16 wks		31% at 16 wks	
Ustekinumab (51)	90 mg	66.4% at 12 wks	78.6% at 28 wks	36.7% at 12 wks	55.6% at 28 wks	10.9% at 12 wks	29.2% at 28 wks
Ustekinumab (52)	90 mg	75.7% at 12 wks	78.5% at 28 wks	50.9% at 12 wks	54.3% at 28 wks	18.2% at 12 wks	29.5% at 28 wks
Secukinumab (135)	150 mg	67% at 12 wks		41.9% at 12 wks		14.4% at 12 wks	
Secukinumab (135)	300 mg	77.1% at 12 wks		54.2% at 12 wks		24.1% at 12 wks	
Ixekizumab (123)	160 mg q2w	89.1% at 12 wks		70.9% at 12 wks		35.3% at 12 wks	
Ixekizumab (123)	160 mg q4w	82.6% at 12 wks		64.6% at 12 wks		33.6% at 12 wks	
Brodalumab (136)	140 mg	60.3% at 12 wks		42.5% at 12 wks		23.3% at 12 wks	
Brodalumab (136)	210 mg	83.3% at 12 wks		70.3% at 12 wks		41.9% at 12 wks	
Bimekizumab (137)	160 mg	81.4% at 12 wks		67.4% at 12 wks		27.9% at 12 wks	
Bimekizumab (137)	320 mg	93.1% at 12 wks		79.1% at 12 wks		55.8% at 12 wks	
Etanercept (135)	50 mg	44% at 12 wks		20.7% at 12 wks		4.3% at 12 wks	
Infliximab (138)	5 mg/kg	75.5% at 10 wks		45.2% at 10 wks			
Adalimumab (139)	40 mg			37% at 12 wks		14% at 12 wks	
Certolizumab pegol (140)	200 mg	61.3% at 12 wks		31.2% at 12 wks			
Golimumab (141)	2 mg/kg	59.2% at 14 wks		39.3% at 14 wks		16.8% at 14 wks	

A recent systematic review of IL-17, IL-17RA, IL-12/23, and IL-23 inhibitors demonstrated that tildrakizumab 100 and 200 mg dosing ranked higher than guselkumab 100 mg, ustekinumab 45 mg, and brodalumab 140 mg dosing in achieving a PASI75 response short-term (142). However, this review had several limitations including the lack of detail on randomization sequence generation, allocation concealment, and blinding in the trials, most of the analyses were indirect comparisons, and the medical histories of patients were not accounted for. Tildrakizumab 100 and 200 mg treatments ranked the lowest for short-term risk of AEs but ranked higher than risankizumab 150 mg in short-term risk of serious AEs. One study compared the safety profiles of tildrakizumab, guselkumab, and risankizumab using phase III clinical trials. The biologic treatments evaluated did not show any significant safety concerns and the overall safety profiles were comparable. The most common AE amongst all evaluated biologic agents was the occurrence of nasopharyngitis/URTIs (143).

A systematic review on the rapidity of onset of action for IL-17 and IL-23 inhibitors for psoriasis demonstrated that the time to onset of action for brodalumab was 2.1–2.6, and 2.2–2.3 weeks for ixekizumab, which were quicker than tildrakizumab (5.6–5.7 weeks), secukinumab (3.0–4.3 weeks), and guselkumab (3.8 weeks) (144). The onset of action was defined by the weighted mean time needed for 25 and 50% of patients to achieve a PASI90 score. A network meta-analysis compared the efficacy of biologic therapies for psoriasis using PASI75, 90, and 100 responses. Specifically, 62 randomized controlled trials were evaluated, and it was determined that tildrakizumab, adalimumab, brodalumab, certolizumab pegol, guselkumab, risankizumab, secukinumab, and ustekinumab were comparable with respect to short-term efficacy and tolerability in comparison to the placebo and methotrexate at 10–16 weeks (145), however, this analysis did not include data beyond 16 weeks of treatment. This study also calculated the numbers needed to treat to benefit/harm (NNTB/NNTH) as the reciprocal of the corresponding risk; thus, the NNTB/NNTH for tildrakizumab vs. placebo was 3 (95% CI: 2–4) and non-significant for tildrakizumab vs. adalimumab in achieving PASI90 clearance during weeks 10–16. Similarly, another Bayesian and Frequentist network meta-analyses using 32 phase III clinical trials demonstrated that brodalumab and ixekizumab had the quickest treatment effects based on PASI75 response at weeks 2, 4, and 8 as well as based on PASI90 and PASI100 scores at weeks 2, 4, 8, and 12. The PASI score changes of tildrakizumab were negligible for the initial 2 weeks of therapy (146). The speed of onset and level of skin improvement between ixekizumab and guselkumab, tildrakizumab, and risankizumab in patients with moderate-to-severe plaque psoriasis were also compared. Matched adjusted indirect comparisons demonstrated that ixekizumab was superior to guselkumab, tildrakizumab, and risankizumab short term (week 2–12) with respect to the onset and clinical efficacy (147). A study comparing the speed of onset and level of improvement between ixekizumab, tildrakizumab, guselkumab, and risankizumab further demonstrated that ixekizumab provided a quicker onset of response and clinical benefit than the IL-23 inhibitors using matched adjusted indirect comparisons from clinical trials (147). Ixekizumab showed favorable results over tildrakizumab based on the data from weeks 2–12 evaluating PASIs 75, 90, and 100 scores. A network meta-analysis comparing the efficacy and safety of risankizumab, guselkumab, tildrakizumab, and ustekinumab to treat moderate-to-severe psoriasis also illustrated that risankizumab 90 and 180 mg doses were more effective than tildrakizumab 5, 25, 100, and 200 mg treatment (148). This study used indirect comparisons and the surface under the cumulative ranking curve. The safety was comparable between all IL-23 inhibitors and placebo.

Another network meta-analysis compared the efficacy and safety of systemic agents including tildrakizumab, guselkumab as well as IL-12/23 and TNF-α (149). The study demonstrated that at class level, all of the interventions including tildrakizumab were significantly more effective than the placebo at reaching PASI90 clearance for chronic plaque psoriasis. However, there was significant difference between the anti-IL-17 agents (brodalumab, ixekizumab, and secukinumab), tildrakizumab and guselkumab when comparing the PASI90 scores. Results from the ranking analysis for quality of life with the surface under the cumulative ranking curve demonstrated that tildrakizumab was inferior to ixekizumab, guselkumab, ustekinumab, and superior to etancercept. Ranking analysis for PGA 0/1 further suggested that tildrakizumab was superior to ixekizumab, secukinnumab, brodalumab, ustekinumab, but was inferior to certolizumab. Ranking analysis for PASI75 further demonstrated that tildrakizumab was superior to ustekinumab but inferior to ixekinumab, secukinumab, and brodlumab. Following the placebo treatment, tildrakizumab demonstrated the best safety profile with regards to the number of adverse events, followed by guselkumab and certolizumab. This network meta-analysis also found no significant differences in serious adverse events between tildrakizumab and other IL-23, IL-17, and 12/23 inhibitors. However, the authors indicated that the number of studies included for tildrakizumab was low, thus this conclusions should be interpreted with caution.

Also, Armstrong et al. recently published an additional network meta-analysis assessing the short and long-term efficacy of biologic treatments in managing moderate-to-severe chronic plaque psoriasis (150). This analysis demonstrates that the short-term PASI90 and 100 response rates (10–16 weeks after study initiation) were higher for ixekizumab, risankizumab, and brodalumab compared to tildrakizumab 200 mg and 100 mg, guselkumab and secukinumab. Guselkumab and secukinumab also had significantly higher response rates compared to tildrakizumab 100 and 200 mg. This analysis did not present data for the long-term efficacy of tildrakiuzmb which was denoted as 48–52 weeks after study initiation.

## Off-Label Use of IL-23 Inhibitors Including Tildrakizumab

IL-23 inhibitors are indicated in conditions other than the moderate-to-severe psoriasis and have been used off label. Guselkumab is approved for the treatment of psoriatic arthritis (PsA), while risankizumab have shown efficacy in treating this disease based on conducted trials (151, 152). IL-23 induces the production of IL-17, which is involved in the inflammatory pathogenesis of psoriatic arthritis (152). However, a 2018 phase II randomized controlled trial demonstrated that risankizumab did not show clinically significant improvements in treating ankylosing spondylitis (153). Guselkumab has been reported as a second- or third-line therapy for HS in 16 cases (154). Guselkumab has also been shown to be effective in patients with alopecia secondary to psoriasis. A case report demonstrated hair regrowth and improvements in areas of psoriatic erythema upon treatment with guselkumab 100 mg (155). In a randomized controlled trial, guselkumab was also shown to improve the palmoplantar pustulosis in 49 patients (156). Phase II and III randomized controlled trials are also being conducted to assess the efficacy of guselkumab for the treatment of inflammatory bowel disease. Notably, two randomized controlled trials along with the transcriptome-wide-RNA-sequence profiling analysis have demonstrated risankizumab to be an effective therapy for Crohn's Disease. Mirikizumab was also shown to be effective and safe in managing ulcerative colitis and Crohn's Disease (157, 158). Risankizumab treatment was recently shown to result in a significant improvement in the treatment of pyoderma gangrenosum at the dose of 150 mg every 8 weeks (159).

Although the focus of this paper is not on the off label uses of tildrakizumab, Table 5 summarizes case studies that utilized tildrakizumab off label, demonstrating potential benefits in other clinical settings. Subcutaneous injections of tildrakizumab 100 mg at weeks 0, 4 and then every 12 week thereafter were reported to improve ulceration in a patient with refractory pyoderma gangrenosum and polymyalgia rheumatica with no recorded AEs (160). Tildrakizumab was also effective in treating a case of PASH syndrome, a rare inflammatory condition characterized by pyoderma gangrenosum, acne, and hidradenitis suppurativa (HS). Specifically, tildrakizumab 100 mg administered at weeks 0 and 4 resulted in a significant reduction in abscess and nodule counts (161). Similar to guselkumab, tildrakizumab was shown to be effective in treating HS in 5 patients. Specifically, 100 mg of tildrakizumab was injected in 5 HS patients at weeks 0 and 4, followed by tildrakizumab 200 mg treatment every 4 weeks thereafter. All patients showed significant improvements in abscess and nodule counts; 4 patients had improvements in the Dermatology Life Quality Index (DLQI), and three patients experienced a reduction in pain symptoms (162). Tildrakizumab was also recently subcutaneously injected for the treatment-resistant lesions of lupus tumidus on the face. Two doses of Tildrakizumab 100 mg significantly improved the facial plaques (163). Another patient with erosive oral lichen planus was treated with tildrakizumab 100 mg injections at weeks 0 and 4, which significantly improved this disease (164). Similarly, near complete resolution of lesions was observed in a patient with recalcitrant lichen planus pemphigoides upon treatment with tildrakizumab 100 mg at weeks 0, 4, and 16 (165). Tildrakizumab has also been employed to induce repigmentation of acrofacial vitiligo. A patient with rapidly progressive vitiligo was treated with tildrakizumab 100 mg at weeks 0, 4, and 12, resulting in a significant repigmentation and improvement of DLQI scores (166). A case of psoriatic nail dystrophy and psoriatic arthritis was also reported, demonstrating a significant improvement in both conditions using tildrakizumab injections at weeks 0 and 4 (167). Treatment of alopecia areata with tildrakizumab 100 mg administered at weeks 0, 4, and 16 has also been reported. Nine patients with alopecia areata were treated with tildrakizumab 100 mg, where 2 patients had a partial response (16–99% improvement) with 2 patients experiencing AEs including URTIs and acne (168). Finally, a patient with recalcitrant lichen planopilaris and frontal fibrosing alopecia demonstrated significant improvement after 4 doses of tildrakizumab 100 mg at weeks 0, 4, and subsequently every 12 weeks. Disease remission was maintained for 13 months (169).

**Table 5:** Clinical cases reported in the literature discussing the off label uses of tildrakizumab.

**Case Report**	**Sample Size**	**Condition**	**Tildrakizumab dosage and regimen**	**Outcome**	**Adverse events**
John and Sinclair (160)	1	Refractory pyoderma gangrenosum of the penis and polymyalgia rheumatica	Tildrakizumab 100 mg; weeks 0, 4, every 12 weeks thereafter	Re-epithelialisation of ulceration, complete resolution	None
Kok et al. (161)	1	Pyoderma gangrenosum, acne and hidradenitis suppurativa (PASH)	Tildrakizumab 100 mg; weeks 0, 4, the tildrakizumab 200 mg every 4 weeks thereafter	Clinical improvement, abscess and nodule count of 5 compared to 68 baseline, DLQI score of 19 compared to 26 baselines	None
Kok et al. (162)	5	Moderate- to-severe hidradenitis suppurativa	Tildrakizumab 100 mg; weeks 0, 4, the tildrakizumab 200 mg every 4 weeks thereafter	All patients demonstrated an improvement, mean reduction of 16.8 (*P* = 0.04) in abscess and nodule count; four patients had DLQI improvement, DLQI, mean difference = 8.0, *P* = 0.46; Three patients had reduction in VAS, mean difference = 1.2, *P* = 0.64	None
Ismail et al. (163)	1	15-year history of treatment-resistant lupus erythematosus tumidus	Tildrakizumab 100 mg, weeks 0, 4, and 16	Improvements in facial plaques	None
Ismail and Sinclair (164)	1	9-month history of biopsy-proven, severe erosive oral lichen planus	Tildrakizumab 100 mg, weeks 0, 4, and 16	complete healing of erosions, with residual fine reticular striations	None
Kerkemeyer et al. (165)	1	15-year history of pruritic lichenoid papules and plaques	Tildrakizumab 100 mg, weeks 0, 4, and 16	Reduction in itch; significant improvement, near-complete clinical resolution after 3 doses	None
Jerjen et al. (166)	1	3-month history of rapidly progressive vitiligo	Tildrakizumab 100 mg; weeks 0, 4, 12, then 3-month intervals	55% reduction in Vitiligo Area Scoring Index, 90% repigmentation in affected areas	None
Ismail et al. (167)	1	Psoriatic nail dystrophy and psoriatic arthritis	Tildrakizumab 100 mg, weeks 0, 4, and 16	Significant improvement; patient noticed reduced time for arthritic pain to ease in the morning	None
Kerkemeyer and Sinclair (168)	10	Alopecia areata	Tildrakizumab 100 mg, weeks 0, 4, and 16	2 patients had a partial response (16–99%); 8 patients had no response; 1 patient with- drew due to no response	Mild; Upper respiratory tract infection, acne
Trindade de Carvalho et al. (169)	1	Recalcitrant lichen planopilaris and frontal fibrosing alopecia	Tildrakizumab 100 mg; weeks 0, 4, every 12 weeks thereafter	Remission and clinical improvements maintained at 13 months	None

## Conclusion

Tildrakizumab is a promising biologic that can be used to treat moderate-to-severe chronic plaque psoriasis. The IL-23 inhibitory mechanism of tildrakizumab plays a central role in hindering the pathogenesis of psoriasis. The reSURFACE trials and the *post-hoc* analyses have demonstrated that tildrakizumab is a reliable biologic therapy. Further head-to-head trials are needed to confirm its efficacy in comparison to other newer biologic agents. Data is also emerging on the off-label use of IL-23 inhibitors making them likely suitable for the treatment of other debilitating skin diseases.

## Author Contributions

FG, FM, LK, MB, YP, RV, MW, CL, and IL performed the literature review and wrote the paper. CL and IL supervised the project. All authors read and approved the final version for submission.

## Conflict of Interest

LK has received honoraria and research grants as a consultant, investigator, speaker, and/or advisory board member for Abbott, Acambis, Allergan, Amgen, Assos Pharma, Astellas Pharma US, Asubio, Berlex Laboratories (Bayer HealthCare Pharmaceuticals), Biogen Idec, Biolife, Biopelle, Breckinridge Pharma, Colbar, Celgene, Centocor, CollaGenex, Combinatrix, Connetics, Coria, Dermik Laboratories, Dow Pharmaceutical Sciences, Dusa, Embil Pharmaceuticals, EOS, Ferndale Laboratories, Galderma, Genentech, GlaxoSmithKline, Health Point, Intendis, Innovail, Johnson & Johnson, Laboratory Skin Care, LEO Pharma, 3M, Medical International Technologies, Merck, Medicis Pharmaceutical, Merz, Nano Bio, Novartis, Nucryst Pharmaceuticals, Obagi, Onset, OrthoNeutrogena, Promius, QLT, PharmaDerm, Pfizer, Quatrix, Serono (Merck Serono International SA), SkinMedica, Stiefel, Sun Pharmaceutical Industries, TolerRx, Triax, Valeant Pharmaceuticals Intl, Warner-Chilcott, and ZAGE. YP has received grant funding and honoraria for services as an investigator, speaker, and member of advisory boards from AbbVie, Amgen, Bausch, Janssen-Ortho, UCB Biopharma, and has received grant funding as an investigator from AnaptysBio, Arcutis biotherapeutics, Asana, Astrazeneca, Baxalta, Baxter, Boehringer Ingelheim, Bond Avillion, Bristol Myers Squibb, Celgene, Dermira, Devonian, Galderma, Genentech, Glaxo Smith Kline, Eli Lilly, Incyte, LEO Pharma, MedImmune, Merck, Novartis, Pfizer, Regeneron, Roche, Sun Pharmaceutical Industries, Serono, Takeda. MB an advisory board member and speaker for AbbVie, Amgen, Leo Pharma, and Janssen; a speaker for Astellas and Merck; and an investigator for AbbVie, Astellas, Amgen, Leo Pharma, Novartis, Janssen, Sun Pharma, Lilly, Pfizer, and Celgene. RV was a speaker for AbbVie, Amgen, Celgene, Galderma, Janssen, Leo Pharma, Novartis, and Pfizer, and was an investigator for AbbVie, Amgen, Celgene, Galderma, Janssen, Leo Pharma, Novartis, Sun Pharmaceutical Industries, Pfizer, Lilly, and Merck. MW reports having received honoraria for ad board participation from Novartis, Sun Pharmaceutical Industries and Pfizer. CL was a consultant, speaker, and advisory board member for Amgen, Pfizer, AbbVie, Janssen, Novartis, and Celgene, and was an investigator for Amgen, Pfizer, AbbVie, Janssen, Lilly, Novartis, and Celgene. YP was a speaker and advisory board member for AbbVie, Amgen, and Janssen, and was an investigator for AbbVie, Amgen, Celgene, Centocor, Lilly, Galderma, Incyte, Sun Pharmaceutical Industries, Janssen, Leo Pharma, Merck, Novartis, Pfizer, and Roche. IL received research grant funding from Novartis, Merck, Abbvie, and Bristol Myers Squibb and honoraria from Janssen, Bausch, Galderma, Novartis, Pfizer, Sun Pharmaceutical Industries, Johnson & Johnson, and Actilion. Topics included in this article were based on, but not limited to, broad discussions at an advisory board meeting, which was sponsored and funded by Sun Pharma Canada, Inc. Consultancy fees were paid to meeting participants (LK, MB, YP, RV, MW, CL, and IL). Sun Pharma Canada, Inc., participated in developing the content for the meeting. The remaining authors declare that the research was conducted in the absence of any commercial or financial relationships that could be construed as a potential conflict of interest.

## Publisher's Note

All claims expressed in this article are solely those of the authors and do not necessarily represent those of their affiliated organizations, or those of the publisher, the editors and the reviewers. Any product that may be evaluated in this article, or claim that may be made by its manufacturer, is not guaranteed or endorsed by the publisher.

## References

[1] Parisi R Symmons DP Griffiths CE Ashcroft DM. Global epidemiology of psoriasis: a systematic review of incidence and prevalence. J Invest Dermatol. (2013) 133:377–85. 10.1038/jid.2012.339 23014338

[2] Chiricozzi A Saraceno R Chimenti MS Guttman-Yassky E Krueger JG. Role of IL-23 in the pathogenesis of psoriasis: a novel potential therapeutic target? Expert Opin Ther Targets. (2014) 18:513–25. 10.1517/14728222.2014.889686 24568095

[3] Martin DA Towne JE Kricorian G Klekotka P Gudjonsson JE Krueger JG. The emerging role of IL-17 in the pathogenesis of psoriasis: preclinical and clinical findings. J Invest Dermatol. (2013) 133:17–26. 10.1038/jid.2012.194 22673731 PMC3568997

[4] Bergboer JGM Zeeuwen P Schalkwijk J. Genetics of psoriasis: evidence for epistatic interaction between skin barrier abnormalities and immune deviation. J Invest Dermatol. (2012) 132:2320–31. 10.1038/jid.2012.167 22622420

[5] Lupardus PJ Garcia KC. The structure of interleukin-23 reveals the molecular basis of p40 subunit sharing with interleukin-12. J Mol Biol. (2008) 382:931–41. 10.1016/j.jmb.2008.07.051 18680750 PMC2666310

[6] Oppmann B Lesley R Blom B Timans JC Xu Y Hunte B. Novel p19 protein engages IL-12p40 to form a cytokine, IL-23, with biological activities similar as well as distinct from IL-12. Immunity. (2000) 13:715–25. 10.1016/S1074-7613(00)00070-4 11114383

[7] Di Meglio P Nestle FO. The role of IL-23 in the immunopathogenesis of psoriasis. F1000 Biol Rep. (2010) 2:40. 10.3410/B2-40 20948793 PMC2950033

[8] Tang C Chen S Qian H Huang W. Interleukin-23: as a drug target for autoimmune inflammatory diseases. Immunology. (2012) 135:112–24. 10.1111/j.1365-2567.2011.03522.x 22044352 PMC3277713

[9] Piskin G Sylva-Steenland RM Bos JD Teunissen MB. In vitro and in situ expression of IL-23 by keratinocytes in healthy skin and psoriasis lesions: enhanced expression in psoriatic skin. J Immunol. (2006) 176:1908–15. 10.4049/jimmunol.176.3.1908 16424222

[10] Smits HH van Beelen AJ Hessle C Westland R de Jong E Soeteman E. Commensal gram-negative bacteria prime human dendritic cells for enhanced IL-23 and IL-27 expression and enhanced Th1 development. Eur J Immunol. (2004) 34:1371–80. 10.1002/eji.200324815 15114670

[11] Liu W Ouyang X Yang J Liu J Li Q Gu Y. AP-1 activated by toll-like receptors regulates expression of IL-23 p19. J Biol Chem. (2009) 284:24006–16. 10.1074/jbc.M109.025528 19592489 PMC2781995

[12] Wilson NJ Boniface K Chan JR McKenzie BS Blumenschein WM Mattson JD. Development, cytokine profile and function of human interleukin 17-producing helper T cells. Nat Immunol. (2007) 8:950–7. 10.1038/ni1497 17676044

[13] McGeachy MJ Bak-Jensen KS Chen Y Tato CM Blumenschein W McClanahan T. TGF-beta and IL-6 drive the production of IL-17 and IL-10 by T cells and restrain T(H)-17 cell-mediated pathology. Nat Immunol. (2007) 8:1390–7. 10.1038/ni1539 17994024

[14] Duvallet E Semerano L Assier E Falgarone G Boissier MC. Interleukin-23: a key cytokine in inflammatory diseases. Ann Med. (2011) 43:503–11. 10.3109/07853890.2011.577093 21585245

[15] Parham C Chirica M Timans J Vaisberg E Travis M Cheung J. A receptor for the heterodimeric cytokine IL-23 is composed of IL-12Rbeta1 and a novel cytokine receptor subunit, IL-23R. J Immunol. (2002) 168:5699–708. 10.4049/jimmunol.168.11.5699 12023369

[16] Levin AA Gottlieb AB. Specific targeting of interleukin-23p19 as effective treatment for psoriasis. J Am Acad Dermatol. (2014) 70:555–61. 10.1016/j.jaad.2013.10.043 24373779

[17] Chen Z Laurence A Kanno Y Pacher-Zavisin M Zhu BM Tato C. Selective regulatory function of Socs3 in the formation of IL-17-secreting T cells. Proc Natl Acad Sci USA. (2006) 103:8137–42. 10.1073/pnas.0600666103 16698929 PMC1459629

[18] Zheng Y Danilenko DM Valdez P Kasman I Eastham-Anderson J Wu J. Interleukin-22, a T(H)17 cytokine, mediates IL-23-induced dermal inflammation and acanthosis. Nature. (2007) 445:648–51. 10.1038/nature05505 17187052

[19] Jeon C Sekhon S Yan D Afifi L Nakamura M Bhutani T. Monoclonal antibodies inhibiting IL-12,−23, and−17 for the treatment of psoriasis. Hum Vaccin Immunother. (2017) 13:2247–59. 10.1080/21645515.2017.1356498 28825875 PMC5647990

[20] Liang SC Tan XY Luxenberg DP Karim R Dunussi-Joannopoulos K Collins M. Interleukin (IL)-22 and IL-17 are coexpressed by Th17 cells and cooperatively enhance expression of antimicrobial peptides. J Exp Med. (2006) 203:2271–9. 10.1084/jem.20061308 16982811 PMC2118116

[21] Ciric B El-behi M Cabrera R Zhang GX Rostami A. IL-23 drives pathogenic IL-17-producing CD8+ T cells. J Immunol. (2009) 182:5296–305. 10.4049/jimmunol.0900036 19380776

[22] Ortega C Fernández AS Carrillo JM Romero P Molina IJ Moreno JC. IL-17-producing CD8+ T lymphocytes from psoriasis skin plaques are cytotoxic effector cells that secrete Th17-related cytokines. J Leukoc Biol. (2009) 86:435–43. 10.1189/JLB.0109046 19487306

[23] Laggner U Di Meglio P Perera GK Hundhausen C Lacy KE Ali N. Identification of a novel proinflammatory human skin-homing Vγ9Vδ2 T cell subset with a potential role in psoriasis. J Immunol. (2011) 187:2783–93. 10.4049/jimmunol.1100804 21813772 PMC3187621

[24] Lin AM Rubin CJ Khandpur R Wang JY Riblett M Yalavarthi S. Mast cells and neutrophils release IL-17 through extracellular trap formation in psoriasis. J Immunol. (2011) 187:490–500. 10.4049/jimmunol.1100123 21606249 PMC3119764

[25] Bielecki P Riesenfeld SJ Hutter JC Torlai Triglia E Kowalczyk MS Ricardo-Gonzalez RR. Skin-resident innate lymphoid cells converge on a pathogenic effector state. Nature. (2021) 592:128–32. 10.1038/s41586-021-03188-w 33536623 PMC8336632

[26] Banaszczyk K. Tildrakizumab in the treatment of psoriasis - literature review. Reumatologia. (2019) 57:234–8. 10.5114/reum.2019.87620 31548750 PMC6753595

[27] Baker BS Swain AF Griffiths CE Leonard JN Fry L Valdimarsson H. Epidermal T lymphocytes and dendritic cells in chronic plaque psoriasis: the effects of PUVA treatment. Clin Exp Immunol. (1985) 61:526–34. 3878241 PMC1577286

[28] Baker BS Griffiths CE Lambert S Powles AV Leonard JN Valdimarsson H. The effects of cyclosporin A on T lymphocyte and dendritic cell sub-populations in psoriasis. Br J Dermatol. (1987) 116:503–10. 10.1111/j.1365-2133.1987.tb05869.x 3495286

[29] Valdimarsson H Baker BS Jónsdóttir I Powles A Fry L. Psoriasis: a T-cell-mediated autoimmune disease induced by streptococcal superantigens? Immunol Today. (1995) 16:145–9. 10.1016/0167-5699(95)80132-4 7718088

[30] Demidem A Taylor JR Grammer SF Streilein JW. T-lymphocyte-activating properties of epidermal antigen-presenting cells from normal and psoriatic skin: evidence that psoriatic epidermal antigen-presenting cells resemble cultured normal langerhans cells. J Invest Dermatol. (1991) 97:454–60. 10.1111/1523-1747.ep12481465 1875046

[31] Lowes MA Kikuchi T Fuentes-Duculan J Cardinale I Zaba LC Haider AS. Psoriasis vulgaris lesions contain discrete populations of Th1 and Th17 T cells. J Invest Dermatol. (2008) 128:1207–11. 10.1038/sj.jid.5701213 18200064

[32] Li J Chen X Liu Z Yue Q Liu H. Expression of Th17 cytokines in skin lesions of patients with psoriasis. J Huazhong Univ Sci Technolog Med Sci. (2007) 27:330–2. 10.1007/s11596-007-0329-1 17641855

[33] Lee E Trepicchio WL Oestreicher JL Pittman D Wang F Chamian F. Increased expression of interleukin 23 p19 and p40 in lesional skin of patients with psoriasis vulgaris. J Exp Med. (2004) 199:125–30. 10.1084/jem.20030451 14707118 PMC1887731

[34] Yawalkar N Tscharner GG Hunger RE Hassan AS. Increased expression of IL-12p70 and IL-23 by multiple dendritic cell and macrophage subsets in plaque psoriasis. J Dermatol Sci. (2009) 54:99–105. 10.1016/j.jdermsci.2009.01.003 19264456

[35] Hvid H Teige I Kvist PH Svensson L Kemp K. TPA induction leads to a Th17-like response in transgenic K14/VEGF mice: a novel in vivo screening model of psoriasis. Int Immunol. (2008) 20:1097–106. 10.1093/intimm/dxn068 18579711

[36] Nakajima K Kanda T Takaishi M Shiga T Miyoshi K Nakajima H. Distinct roles of IL-23 and IL-17 in the development of psoriasis-like lesions in a mouse model. J Immunol. (2011) 186:4481–9. 10.4049/jimmunol.1000148 21346238

[37] van der Fits L Mourits S Voerman JS Kant M Boon L Laman JD. Imiquimod-induced psoriasis-like skin inflammation in mice is mediated via the IL-23/IL-17 axis. J Immunol. (2009) 182:5836–45. 10.4049/jimmunol.0802999 19380832

[38] Tonel G Conrad C Laggner U Di Meglio P Grys K McClanahan TK. Cutting edge: A critical functional role for IL-23 in psoriasis. J Immunol. (2010) 185:5688–91. 10.4049/jimmunol.1001538 20956338 PMC3776381

[39] Rizzo HL Kagami S Phillips KG Kurtz SE Jacques SL Blauvelt A. IL-23-mediated psoriasis-like epidermal hyperplasia is dependent on IL-17A. J Immunol. (2011) 186:1495–502. 10.4049/jimmunol.1001001 21172868

[40] Boutet MA Nerviani A Gallo Afflitto G Pitzalis C. Role of the IL-23/IL-17 axis in psoriasis and psoriatic arthritis: the clinical importance of its divergence in skin and joints. Int J Mol Sci. (2018) 19:530. 10.3390/ijms19020530 PMC5855752 29425183

[41] Brembilla NC Senra L Boehncke WH. The IL-17 family of cytokines in psoriasis: IL-17A and beyond. Front Immunol. (2018) 9:1682. 10.3389/fimmu.2018.01682 30127781 PMC6088173

[42] Harper EG Guo C Rizzo H Lillis JV Kurtz SE Skorcheva I. Th17 cytokines stimulate CCL20 expression in keratinocytes *in vitro* and *in vivo*: implications for psoriasis pathogenesis. J Invest Dermatol. (2009) 129:2175–83. 10.1038/jid.2009.65 19295614 PMC2892172

[43] Ramirez-Carrozzi V Sambandam A Luis E Lin Z Jeet S Lesch J. IL-17C regulates the innate immune function of epithelial cells in an autocrine manner. Nat Immunol. (2011) 12:1159–66. 10.1038/ni.2156 21993848

[44] McGeachy MJ Chen Y Tato CM Laurence A Joyce-Shaikh B Blumenschein WM. The interleukin 23 receptor is essential for the terminal differentiation of interleukin 17-producing effector T helper cells in vivo. Nat Immunol. (2009) 10:314–24. 10.1038/ni.1698 19182808 PMC2945605

[45] Nestle FO Conrad C Tun-Kyi A Homey B Gombert M Boyman O. Plasmacytoid predendritic cells initiate psoriasis through interferon-alpha production. J Exp Med. (2005) 202:135–43. 10.1084/jem.20050500 15998792 PMC2212894

[46] Mease PJ. Inhibition of interleukin-17, interleukin-23 and the TH17 cell pathway in the treatment of psoriatic arthritis and psoriasis. Curr Opin Rheumatol. (2015) 27:127–33. 10.1097/BOR.0000000000000147 25599143

[47] Puig L. The role of IL 23 in the treatment of psoriasis. Expert Rev Clin Immunol. (2017) 13:525–34. 10.1080/1744666X.2017.1292137 28165883

[48] Benson JM Sachs CW Treacy G Zhou H Pendley CE Brodmerkel CM. Therapeutic targeting of the IL-12/23 pathways: generation and characterization of ustekinumab. Nat Biotechnol. (2011) 29:615–24. 10.1038/nbt.1903 21747388

[49] Griffiths CE Strober BE van de Kerkhof P Ho V Fidelus-Gort R Yeilding N. Comparison of ustekinumab and etanercept for moderate-to-severe psoriasis. N Engl J Med. (2010) 362:118–28. 10.1056/NEJMoa0810652 20071701

[50] Lebwohl M Leonardi C Griffiths CE Prinz JC Szapary PO Yeilding N. Long-term safety experience of ustekinumab in patients with moderate-to-severe psoriasis (part I of II): results from analyses of general safety parameters from pooled phase 2 and 3 clinical trials. J Am Acad Dermatol. (2012) 66:731–41. 10.1016/j.jaad.2011.06.011 21930328

[51] Leonardi CL Kimball AB Papp KA Yeilding N Guzzo C Wang Y. Efficacy and safety of ustekinumab, a human interleukin-12/23 monoclonal antibody, in patients with psoriasis: 76-week results from a randomised, double-blind, placebo-controlled trial (PHOENIX 1). Lancet. (2008) 371:1665–74. 10.1016/S0140-6736(08)60725-4 18486739

[52] Papp KA Langley RG Lebwohl M Krueger GG Szapary P Yeilding N. Efficacy and safety of ustekinumab, a human interleukin-12/23 monoclonal antibody, in patients with psoriasis: 52-week results from a randomised, double-blind, placebo-controlled trial (PHOENIX 2). Lancet. (2008) 371:1675–84. 10.1016/S0140-6736(08)60726-6 18486740

[53] Fotiadou C Lazaridou E Sotiriou E Ioannides D. Targeting IL-23 in psoriasis: current perspectives. Psoriasis. (2018) 8:1–5. 10.2147/PTT.S98893 29441315 PMC5804022

[54] Ibler E Gordon KB. IL-23 inhibitors for moderate-to-severe psoriasis. Semin Cutan Med Surg. (2018) 37:158–62. 10.12788/j.sder.2018.047 30215632

[55] Xu S Zhang X Pan M Shuai Z Xu S Pan F. Treatment of plaque psoriasis with IL-23p19 blockers: a systematic review and meta-analysis. Int Immunopharmacol. (2019) 75:105841. 10.1016/j.intimp.2019.105841 31465912

[56] Li SJ Perez-Chada LM Merola JF. TNF inhibitor-induced psoriasis: proposed algorithm for treatment and management. J Psoriasis Psoriatic Arthritis. (2019) 4:70–80. 10.1177/2475530318810851 31093599 PMC6513344

[57] Liau MM Oon HH. Therapeutic drug monitoring of biologics in psoriasis. Biologics. (2019) 13:127–32. 10.2147/BTT.S188286 31308623 PMC6613538

[58] Lee EB Amin M Bhutani T Wu JJ. Emerging therapies in psoriasis: a systematic review. Cutis. (2018) 101:5–9. 29718027

[59] Tausend W Downing C Tyring S. Systematic review of interleukin-12, interleukin-17, and interleukin-23 pathway inhibitors for the treatment of moderate-to-severe chronic plaque psoriasis: ustekinumab, briakinumab, tildrakizumab, guselkumab, secukinumab, ixekizumab, and brodalumab. J Cutan Med Surg. (2014) 18:156–69. 10.2310/7750.2013.13125 24800703

[60] Tonini A Gualtieri B Panduri S Romanelli M Chiricozzi A. A new class of biologic agents facing the therapeutic paradigm in psoriasis: anti-IL-23 agents. Expert Opin Biol Ther. (2018) 18:135–48. 10.1080/14712598.2018.1398729 29103330

[61] Yiu ZZ Warren RB. Efficacy and safety of emerging immunotherapies in psoriasis. Immunotherapy. (2015) 7:119–33. 10.2217/imt.14.101 25713988

[62] Government of Canada. Drug Product Database Online Query From Health Canada. Available online at: https://health-products.canada.ca/dpd-bdpp/index-eng.jsp (accessed February 25, 2021).

[63] US Food and Drug Administration. Food US, Drug Administration. Available online at: https://www.accessdata.fda.gov/scripts/cder/daf/ (accessed February 25, 2021).

[64] European Medicine Agency. Medicines. Available online at: https://www.ema.europa.eu/en/medicines (accessed February 25, 2021).

[65] Langley RG Lebwohl M Krueger GG Szapary PO Wasfi Y Chan D. Long-term efficacy and safety of ustekinumab, with and without dosing adjustment, in patients with moderate-to-severe psoriasis: results from the PHOENIX 2 study through 5 years of follow-up. Br J Dermatol. (2015) 172:1371–83. 10.1111/bjd.13469 25307931

[66] Papp KA Griffiths CE Gordon K Lebwohl M Szapary PO Wasfi Y. Long-term safety of ustekinumab in patients with moderate-to-severe psoriasis: final results from 5 years of follow-up. Br J Dermatol. (2013) 168:844–54. 10.1111/bjd.12214 23301632

[67] Kimball AB Gordon KB Fakharzadeh S Yeilding N Szapary PO Schenkel B. Long-term efficacy of ustekinumab in patients with moderate-to-severe psoriasis: results from the PHOENIX 1 trial through up to 3 years. Br J Dermatol. (2012) 166:861–72. 10.1111/j.1365-2133.2012.10901.x 22356258

[68] Gottlieb AB Leonardi C Kerdel F Mehlis S Olds M Williams DA. Efficacy and safety of briakinumab vs. etanercept and placebo in patients with moderate to severe chronic plaque psoriasis. Br J Dermatol. (2011) 165:652–60. 10.1111/j.1365-2133.2011.10418.x 21574983

[69] Kimball AB Gordon KB Langley RG Menter A Chartash EK Valdes J. Safety and efficacy of ABT-874, a fully human interleukin 12/23 monoclonal antibody, in the treatment of moderate to severe chronic plaque psoriasis: results of a randomized, placebo-controlled, phase 2 trial. Arch Dermatol. (2008) 144:200–7. 10.1001/archdermatol.2007.63 18283176

[70] Markham A. Guselkumab: first global approval. Drugs. (2017) 77:1487–92. 10.1007/s40265-017-0800-7 28819723

[71] Al-Salama ZT Scott LJ. Guselkumab: a review in moderate to severe plaque psoriasis. Am J Clin Dermatol. (2018) 19:907–18. 10.1007/s40257-018-0406-1 30467781

[72] Blauvelt A Papp KA Griffiths CE Randazzo B Wasfi Y Shen YK. Efficacy and safety of guselkumab, an anti-interleukin-23 monoclonal antibody, compared with adalimumab for the continuous treatment of patients with moderate to severe psoriasis: results from the phase III, double-blinded, placebo- and active comparator-controlled VOYAGE 1 trial. J Am Acad Dermatol. (2017) 76:405–17. 10.1016/j.jaad.2016.11.041 28057360

[73] Sofen H Smith S Matheson RT Leonardi CL Calderon C Brodmerkel C. Guselkumab (an IL-23-specific mAb) demonstrates clinical and molecular response in patients with moderate-to-severe psoriasis. J Allergy Clin Immunol. (2014) 133:1032–40. 10.1016/j.jaci.2014.01.025 24679469

[74] Reich K Armstrong AW Foley P Song M Wasfi Y Randazzo B. Efficacy and safety of guselkumab, an anti-interleukin-23 monoclonal antibody, compared with adalimumab for the treatment of patients with moderate to severe psoriasis with randomized withdrawal and retreatment: results from the phase III, double-blind, placebo- and active comparator-controlled VOYAGE 2 trial. J Am Acad Dermatol. (2017) 76:418–31. 10.1016/j.jaad.2016.11.042 28057361

[75] Langley RG Tsai TF Flavin S Song M Randazzo B Wasfi Y. Efficacy and safety of guselkumab in patients with psoriasis who have an inadequate response to ustekinumab: results of the randomized, double-blind, phase III NAVIGATE trial. Br J Dermatol. (2018) 178:114–23. 10.1111/bjd.15750 28635018

[76] Du Jardin KG Hurtado Lopez P Lange M McCool R Maeso Naval S Quickert S. A systematic literature review and bucher indirect comparison: tildrakizumab versus guselkumab. J Health Econ Outcomes Res. (2020) 7:123–9. 10.36469/jheor.2020.13671 32766377 PMC7398610

[77] Singh S Kroe-Barrett RR Canada KA Zhu X Sepulveda E Wu H. Selective targeting of the IL23 pathway: generation and characterization of a novel high-affinity humanized anti-IL23A antibody. MABS. (2015) 7:778–91. 10.1080/19420862.2015.1032491 25905918 PMC4622456

[78] Reich K Gooderham M Thaçi D Crowley JJ Ryan C Krueger JG. Risankizumab compared with adalimumab in patients with moderate-to-severe plaque psoriasis (IMMvent): a randomised, double-blind, active-comparator-controlled phase 3 trial. Lancet. (2019) 394:576–86. 10.1016/S0140-6736(19)30952-3 31280967

[79] Gordon KB Strober B Lebwohl M Augustin M Blauvelt A Poulin Y. Efficacy and safety of risankizumab in moderate-to-severe plaque psoriasis (UltIMMa-1 and UltIMMa-2): results from two double-blind, randomised, placebo-controlled and ustekinumab-controlled phase 3 trials. Lancet. (2018) 392:650–61. 10.1016/S0140-6736(18)31713-6 30097359

[80] Blauvelt A Leonardi CL Gooderham M Papp KA Philipp S Wu JJ. Efficacy and safety of continuous risankizumab therapy vs treatment withdrawal in patients with moderate to severe plaque psoriasis: a phase 3 randomized clinical trial. JAMA Dermatol. (2020) 156:649–58. 10.1001/jamadermatol.2020.0723 32267471 PMC7142813

[81] Visvanathan S Baum P Vinisko R Schmid R Flack M Lalovic B. Psoriatic skin molecular and histopathologic profiles after treatment with risankizumab versus ustekinumab. J Allergy Clin Immunol. (2019) 143:2158–69. 10.1016/j.jaci.2018.11.042 30578873

[82] Reich K Rich P Maari C Bissonnette R Leonardi C Menter A. Efficacy and safety of mirikizumab (LY3074828) in the treatment of moderate-to-severe plaque psoriasis: results from a randomized phase II study. Br J Dermatol. (2019) 181:88–95. 10.1111/bjd.17628 30734266

[83] Yiu ZZ Warren RB. The potential utility of tildrakizumab: an interleukin-23 inhibitor for the treatment of psoriasis. Expert Opin Investig Drugs. (2017) 26:243–9. 10.1080/13543784.2017.1274734 28042732

[84] Frampton JE. Tildrakizumab: A review in moderate-to-severe plaque psoriasis. Am J Clin Dermatol. (2019) 20:295–306. 10.1007/s40257-019-00435-9 30924030

[85] Tildrakizumab (Ilumya) - another IL-23 antagonist for psoriasis. Med Lett Drugs Ther. (2019) 60:4–6. 30681661

[86] Cline A Feldman SR. The perceived promise of p19 inhibitors. Br J Dermatol. (2018) 179:556–7. 10.1111/bjd.16941 30222877

[87] Gupta AK Versteeg SG Abramovits W Vincent KD. Ilumya® (tildrakizumab): a newly approved interluekin-23 antagonist for the treatment of plaque psoriasis. Skinmed. (2018) 16:321–24. 30413226

[88] Kaplon H Reichert JM. Antibodies to watch in 2018. MAbs. (2018) 10:183–203. 10.1080/19420862.2018.1415671 29300693 PMC5825203

[89] Kaplon H Reichert JM. Antibodies to watch in 2019. MAbs. (2019) 11:219–38. 10.1080/19420862.2018.1556465 30516432 PMC6380461

[90] Mansouri Y Goldenberg G. New systemic therapies for psoriasis. Cutis. (2015) 95:155–60. 25844781

[91] Mospan C Mospan G Byland E Whitaker WB Xiong L Dunlap J. Drug updates and approvals: 2018 in review. Nurse Pract. (2018) 43:23–32. 10.1097/01.NPR.0000547548.63764.6b 30379711

[92] Paton DM Tildrakizumab: monoclonal antibody against IL-23p19 for moderate to severe psoriasis. Drugs Today. (2018) 54:433–44. 10.1358/dot.2018.54.7.2866117 30090880

[93] Reichert JM. Antibodies to watch in 2015. MAbs. (2015) 7:1–8. 10.4161/19420862.2015.988944 25484055 PMC4622967

[94] Yang EJ Beck KM Liao W. Tildrakizumab-asmn: what's in a name? Am J Clin Dermatol. (2018) 19:291–2. 10.1007/s40257-018-0357-6 29687361

[95] Services DH. BLA Multi-disciplinary Review and Evaluation - BLA 761067 ILUMYA (Tildrakizumab) Injection. Carlow: Food and Drug Administration (2018).

[96] Beck KM Sanchez IM Yang EJ Liao W. Profile of tildrakizumab-asmn in the treatment of moderate-to-severe plaque psoriasis: evidence to date. Psoriasis. (2018) 8:49–58. 10.2147/PTT.S146640 30214892 PMC6120577

[97] Choy M. Pharmaceutical approval update. P T. (2018) 43:461–2. 30100686 PMC6065498

[98] Computational Fluid Dynamics Research. BLA Multi-Disciplinary Review and Evaluation - BLA761061 TREMFYA (Guselkumab) Injection. Computational Fluid Dynamics Research (2016).

[99] Merck & Co. ILUMYA™ (Tildrakizumab-asmn) Injection. U.S. Approval: 2018. Carlow: Merck & Co (2018).

[100] Menter A Strober BE Kaplan DH Kivelevitch D Prater EF Stoff B. Joint AAD-NPF guidelines of care for the management and treatment of psoriasis with biologics. J Am Acad Dermatol. (2019) 80:1029–72. 10.1016/j.jaad.2018.11.057 30772098

[101] Kopp T Riedl E Bangert C Bowman EP Greisenegger E Horowitz A. Clinical improvement in psoriasis with specific targeting of interleukin-23. Nature. (2015) 521:222–6. 10.1038/nature14175 25754330

[102] Papp K Thaci D Reich K Riedl E Langley RG Krueger JG. Tildrakizumab (MK-3222), an anti-interleukin-23p19 monoclonal antibody, improves psoriasis in a phase IIb randomized placebo-controlled trial. Br J Dermatol. (2015) 173:930–9. 10.1111/bjd.13932 26042589

[103] Reich K Papp KA Blauvelt A Tyring SK Sinclair R Thaci D. Tildrakizumab versus placebo or etanercept for chronic plaque psoriasis (reSURFACE 1 and reSURFACE 2): results from two randomised controlled, phase 3 trials. Lancet. (2017) 390:276–88. 10.1016/S0140-6736(17)31279-5 28596043

[104] Markham A. Tildrakizumab: first global approval. Drugs. (2018) 78:845–9. 10.1007/s40265-018-0917-3 29752706

[105] Thaci D Piaserico S Warren RB Gupta AK Cantrell W Draelos Z. Five-year efficacy and safety of tildrakizumab in patients with moderate to severe psoriasis who respond at week 28: pooled analyses of two randomised phase 3 clinical trials (reSURFACE 1 and reSURFACE 2). Br J Dermatol. (2021). 10.1111/bjd.19866 33544883

[106] Poulin Y Ramon M Rosoph L Weisman J Mendelsohn AM Parno J. Efficacy of tildrakizumab by patient demographic and disease characteristics across a phase 2b and 2 phase 3 trials in patients with moderate-to-severe chronic plaque psoriasis. J Eur Acad Dermatol Venereol. (2020) 34:1500–9. 10.1111/jdv.16187 31919889

[107] Menter MA Murakawa GJ Glover H Mendelsohn AM Parno J Rozzo SJ. Clearance of head and neck involvement in plaque psoriasis with tildrakizumab treatment in the phase 3 reSURFACE 1 study. J Eur Acad Dermatol Venereol. (2020) 34:e803–5. 10.1111/jdv.16648 32432798 PMC7953895

[108] Elewski B Menter A Crowley J Tyring S Zhao Y Lowry S. Sustained and continuously improved efficacy of tildrakizumab in patients with moderate-to-severe plaque psoriasis. J Dermatolog Treat. (2020) 31:763–8. 10.1080/09546634.2019.1640348 31268369

[109] Gordon KB Reich K Crowley JJ Korman NJ Murphy FT Poulin Y. Disease activity and treatment efficacy using patient-level psoriasis area and severity index scores from tildrakizumab phase 3 clinical trials. J Dermatolog Treat. (2020) 1–10. 10.1080/09546634.2020.1747590 32349565

[110] Warren RB Carrascosa JM Fumero E Schoenenberger A Lebwohl MG Szepietowski JC. Time to relapse after tildrakizumab withdrawal in patients with moderate-to-severe psoriasis who were responders at week 28: *post hoc* analysis through 64 weeks from reSURFACE 1 trial. J Eur Acad Dermatol Venereol. (2021) 35:919–27. 10.1111/jdv.16964 32979235

[111] Carrico J Zhao Y Jia X Brodtkorb TH Mendelsohn A Lowry S. The budget impact of introducing tildrakizumab to a united states health plan for managing moderate-to-severe plaque psoriasis. Pharmacoecon Open. (2020) 4:669–77. 10.1007/s41669-020-00208-9 32219733 PMC7688845

[112] Jia X Zhao Y Carrico J Brodtkorb TH Mendelsohn AM Lowry S. Cost-effectiveness of tildrakizumab for the treatment of moderate-to-severe psoriasis in the United States. J Dermatolog Treat. (2020) 1–9. 10.1080/09546634.2020.1773382 32602762

[113] Wu JJ Jia X Zhao Y Carrico J Brodtkorb TH Mendelsohn A. Comparative cost-effectiveness of tildrakizumab and other commonly used treatments for moderate-to-severe psoriasis. J Dermatolog Treat. (2020) 1–8. 10.1080/09546634.2019.1698700 32233828

[114] Jauslin P Kulkarni P Li H Vatakuti S Hussain A Wenning L. Population-Pharmacokinetic modeling of tildrakizumab (MK-3222), an anti-interleukin-23-p19 monoclonal antibody, in healthy volunteers and subjects with psoriasis. Clin Pharmacokinet. (2019) 58:1059–68. 10.1007/s40262-019-00743-7 30915660

[115] Zhang TT Ma J Durbin KR Montavon T Lacy SE Jenkins GJ. Determination of IL-23 pharmacokinetics by highly sensitive accelerator mass spectrometry subsequent modeling to project il-23 suppression in psoriasis patients treated with anti-IL-23 antibodies. AAPS J. (2019) 21:82. 10.1208/s12248-019-0352-8 31250228

[116] Zandvliet A Glasgow S Horowitz A Montgomery D Marjason J Mehta A. Tildrakizumab, a novel anti-IL-23 monoclonal antibody, is unaffected by ethnic variability in Caucasian, Chinese, Japanese subjects. Int J Clin Pharmacol Ther. (2015) 53:139–46. 10.5414/CP202176 25546162

[117] Khalilieh S Hodsman P Xu C Tzontcheva A Glasgow S Montgomery D. Pharmacokinetics of tildrakizumab (MK-3222), an Anti-IL-23 monoclonal antibody, after intravenous or subcutaneous administration in healthy subjects. Basic Clin Pharmacol Toxicol. (2018) 123:294–300. 10.1111/bcpt.13001 29510001

[118] Pithadia DJ Reynolds KA Lee EB Liao W Wu JJ. Tildrakizumab in the treatment of psoriasis: latest evidence and place in therapy. Ther Adv Chronic Dis. (2019) 10:2040622319865658. 10.1177/2040622319865658 31448070 PMC6691657

[119] Khalilieh S Hussain A Montgomery D Levine V Shaw PM Bodrug I. Effect of tildrakizumab (MK-3222), a high affinity, selective anti-IL23p19 monoclonal antibody, on cytochrome P450 metabolism in subjects with moderate to severe psoriasis. Br J Clin Pharmacol. (2018) 84:2292–302. 10.1111/bcp.13670 29926968 PMC6138510

[120] Kimball AB Kerbusch T van Aarle F Kulkarni P Li Q Blauvelt A. Assessment of the effects of immunogenicity on the pharmacokinetics, efficacy and safety of tildrakizumab. Br J Dermatol. (2020) 182:180–9. 10.1111/bjd.17918 30916381 PMC6972989

[121] Nogueira M Warren RB Torres T. Risk of tuberculosis reactivation with interleukin (IL)-17 and IL-23 inhibitors in psoriasis - time for a paradigm change. J Eur Acad Dermatol Venereol. (2021) 35:824–34. 10.1111/jdv.16866 32790003

[122] Mui UN Patel RR Vangipuram R Tyring SK. Tildrakizumab for moderate-to-severe plaque psoriasis. Skin Therapy Lett. (2019) 24:1–4. 31801012

[123] Gordon KB Blauvelt A Papp KA Langley RG Luger T Ohtsuki M. Phase 3 trials of ixekizumab in moderate-to-severe plaque psoriasis. N Engl J Med. (2016) 375:345–56. 10.1056/NEJMoa1512711 27299809

[124] Gooderham M Elewski BE Pariser DM Sofen H Mendelsohn AM Rozzo SJ. Incidence of serious gastrointestinal events among tildrakizumab-treated patients with psoriasis: letter to the editor. J Eur Acad Dermatol Venereol. (2019) 33:e350–2. 10.1111/jdv.15643 31033068 PMC6850306

[125] Blauvelt A Reich K Papp KA Kimball AB Gooderham M Tyring SK. Safety of tildrakizumab for moderate-to-severe plaque psoriasis: pooled analysis of three randomized controlled trials. Br J Dermatol. (2018) 179:615–22. 10.1111/bjd.16724 29742274

[126] Gisondi P Ferrazzi A Girolomoni G. Metabolic comorbidities and psoriasis. Acta Dermatovenerol Croat. (2010) 18:297–304. 21251450

[127] Lebwohl MG Leonardi CL Mehta NN Gottlieb AB Mendelsohn AM Parno J. Tildrakizumab efficacy and safety are not altered by metabolic syndrome status in patients with psoriasis: *post hoc* analysis of 2 phase 3 randomized controlled studies (reSURFACE 1 and reSURFACE 2). J Am Acad Dermatol. (2020) 82:519–22. 10.1016/j.jaad.2019.09.042 31563641

[128] Menter MA Mehta NN Lebwohl MG Gottlieb AB Mendelsohn AM Rozzo SJ. The effect of tildrakizumab on cardiometabolic risk factors in psoriasis by metabolic syndrome status: *post hoc* analysis of two phase 3 trials (ReSURFACE 1 and ReSURFACE 2). J Drugs Dermatol. (2020) 19:703–8. 10.36849/JDD.2020.5337 32845115

[129] Bissonnette R Fernández-Peñas P Puig L Mendelsohn AM Rozzo SJ Menter A. Incidence of cardiovascular events among tildrakizumab-treated patients with moderate-to-severe plaque psoriasis: pooled data from three large randomised clinical trials. J Eur Acad Dermatol Venereol. (2020) 34:e21–4. 10.1111/jdv.15866 31403725

[130] Wang JX Norman RJ Wilcox AJ. Incidence of spontaneous abortion among pregnancies produced by assisted reproductive technology. Hum Reprod. (2004) 19:272–7. 10.1093/humrep/deh078 14747166

[131] Haycraft K DiRuggiero D Rozzo SJ Mendelsohn AM Bhutani T. Outcomes of pregnancies from the tildrakizumab phase I-III clinical development programme. Br J Dermatol. (2020) 183:184–6. 10.1111/bjd.18897 31995637

[132] National Library of Medicine. Tildrakizumab, Drugs and Lactation Database (LactMed). Bethesda, MD: National Library of Medicine (2006).

[133] Yeung J Gooderham MJ Grewal P Hong CH Lansang P Papp KA. Management of plaque psoriasis with biologic therapies in women of child-bearing potential consensus paper. J Cutan Med Surg. (2020) 24:3s−14s. 10.1177/1203475420928376 32500730

[134] Santostefano M Herzyk D Montgomery D Wolf J. Nonclinical safety of tildrakizumab, a humanized anti-IL-23p19 monoclonal antibody, in nonhuman primates. Regul Toxicol Pharmacol. (2019) 108:104476. 10.1016/j.yrtph.2019.104476 31536773

[135] Langley RG Elewski BE Lebwohl M Reich K Griffiths CE Papp K. Secukinumab in plaque psoriasis–results of two phase 3 trials. N Engl J Med. (2014) 371:326–38. 10.1056/NEJMoa1314258 25007392

[136] Papp KA Reich K Paul C Blauvelt A Baran W Bolduc C. A prospective phase III, randomized, double-blind, placebo-controlled study of brodalumab in patients with moderate-to-severe plaque psoriasis. Br J Dermatol. (2016) 175:273–86. 10.1111/bjd.14493 26914406

[137] Papp KA Merola JF Gottlieb AB Griffiths CEM Cross N Peterson L. Dual neutralization of both interleukin 17A and interleukin 17F with bimekizumab in patients with psoriasis: results from BE ABLE 1, a 12-week randomized, double-blinded, placebo-controlled phase 2b trial. J Am Acad Dermatol. (2018) 79:277–86.e10. 10.1016/j.jaad.2018.03.037 29609013

[138] Menter A Feldman SR Weinstein GD Papp K Evans R Guzzo C. A randomized comparison of continuous vs. intermittent infliximab maintenance regimens over 1 year in the treatment of moderate-to-severe plaque psoriasis. J Am Acad Dermatol. (2007) 56:31.e1–15. 10.1016/j.jaad.2006.07.017 17097378

[139] Menter A Tyring SK Gordon K Kimball AB Leonardi CL Langley RG. Adalimumab therapy for moderate to severe psoriasis: a randomized, controlled phase III trial. J Am Acad Dermatol. (2008) 58:106–15. 10.1016/j.jaad.2007.09.010 17936411

[140] Lebwohl M Blauvelt A Paul C Sofen H Wegłowska J Piguet V. Certolizumab pegol for the treatment of chronic plaque psoriasis: results through 48 weeks of a phase 3, multicenter, randomized, double-blind, etanercept- and placebo-controlled study (CIMPACT). J Am Acad Dermatol. (2018) 79:266–76.e5. 10.1016/j.jaad.2018.04.013 29660425

[141] Kavanaugh A Husni ME Harrison DD Kim L Lo KH Leu JH. Safety and efficacy of intravenous golimumab in patients with active psoriatic arthritis: results through week twenty-four of the GO-VIBRANT Study. Arthritis Rheumatol. (2017) 69:2151–61. 10.1002/art.40226 28805045 PMC5765449

[142] Bai F Li GG Liu Q Niu X Li R Ma H. Short-Term efficacy and safety of IL-17, IL-12/23, and IL-23 inhibitors brodalumab, secukinumab, ixekizumab, ustekinumab, guselkumab, tildrakizumab, and risankizumab for the treatment of moderate to severe plaque psoriasis: a systematic review and network meta-analysis of randomized controlled trials. J Immunol Res. (2019) 2019:2546161. 10.1155/2019/2546161 31583255 PMC6754904

[143] Crowley JJ Warren RB Cather JC. Safety of selective IL-23p19 inhibitors for the treatment of psoriasis. J Eur Acad Dermatol Venereol. (2019) 33:1676–84. 10.1111/jdv.15653 31054215 PMC6771721

[144] Egeberg A Andersen YMF Halling-Overgaard AS Alignahi F Thyssen JP Burge R. Systematic review on rapidity of onset of action for interleukin-17 and interleukin-23 inhibitors for psoriasis. J Eur Acad Dermatol Venereol. (2020) 34:39–46. 10.1111/jdv.15920 31465593

[145] Mahil SK Ezejimofor MC Exton LS Manounah L Burden AD Coates LC. Comparing the efficacy and tolerability of biologic therapies in psoriasis: an updated network meta-analysis. Br J Dermatol. (2020) 183:638–49. 10.1111/bjd.19325 32562551

[146] Warren RB See K Burge R Zhang Y Brnabic A Gallo G. Rapid response of biologic treatments of moderate-to-severe plaque psoriasis: a comprehensive investigation using bayesian and frequentist network meta-analyses. Dermatol Ther. (2020) 10:73–86. 10.1007/s13555-019-00337-y PMC6994587 31686337

[147] Gottlieb AB Saure D Wilhelm S Dossenbach M Schuster C Smith SD. Indirect comparisons of ixekizumab versus three interleukin-23 p19 inhibitors in patients with moderate-to-severe plaque psoriasis - efficacy findings up to week 12. J Dermatolog Treat. (2020) 1–8. 10.1080/09546634.2020.1747592 32299269

[148] Shi J Xu J Chen Y. A network meta-analysis for the comparison of efficacy and safety of interleukin (IL)-23 targeted drugs in the treatment of moderate to severe psoriasis. Dermatol Ther. (2020) 33:e13802. 10.1111/dth.13802 32521069

[149] Sbidian E Chaimani A Garcia-Doval I Do G Hua C Mazaud C. Chosidow, Systemic pharmacological treatments for chronic plaque psoriasis: a network meta-analysis. Cochrane Database Syst Rev. (2017) 12:CD011535. 10.1002/14651858.CD011535.pub2 29271481 PMC6486272

[150] Armstrong AW Soliman AM Betts KA Wang Y Gao Y Puig L. Comparative efficacy and relative ranking of biologics and oral therapies for moderate-to-severe plaque psoriasis: a network meta-analysis. Dermatol Ther. (2021) 11:885–905. 10.1007/s13555-021-00511-1 PMC8163943 33788177

[151] Deodhar A Helliwell PS Boehncke WH Kollmeier AP Hsia EC Subramanian RA. Guselkumab in patients with active psoriatic arthritis who were biologic-naive or had previously received TNFα inhibitor treatment (DISCOVER-1): a double-blind, randomised, placebo-controlled phase 3 trial. Lancet. (2020) 395:1115–25. 10.1016/S0140-6736(20)30265-8 32178765

[152] Sakkas LI Zafiriou E Bogdanos DP. Mini review: new treatments in psoriatic arthritis. Focus on the IL-23/17 axis. Front Pharmacol. (2019) 10:872. 10.3389/fphar.2019.00872 31447673 PMC6691125

[153] Baeten D Østergaard M Wei JC Sieper J Järvinen P Tam LS Salvarani C. Risankizumab, an IL-23 inhibitor, for ankylosing spondylitis: results of a randomised, double-blind, placebo-controlled, proof-of-concept, dose-finding phase 2 study. Ann Rheum Dis. (2018) 77:1295–302. 10.1136/annrheumdis-2018-213328 29945918 PMC6104676

[154] Montero-Vilchez T Martinez-Lopez A Salvador-Rodriguez L Arias-Santiago S Molina-Leyva A. The use of guselkumab 100 mg every 4 weeks on patients with hidradenitis suppurativa and a literature review. Dermatol Ther. (2020) 33:e13456. 10.1111/dth.13456 32319172

[155] Hosokawa Y Hamada T Ashida H Ikeda M. Effective treatment with guselkumab for psoriatic alopecia as paradoxical reaction. J Dermatol. (2019) 46:e302–3. 10.1111/1346-8138.14842 30861165

[156] Terui T Kobayashi S Okubo Y Murakami M Hirose K Kubo H. Efficacy and safety of guselkumab, an anti-interleukin 23 monoclonal antibody, for palmoplantar pustulosis: a randomized clinical trial. JAMA Dermatol. (2018) 154:309–16. 10.1001/jamadermatol.2017.5937 29417135 PMC5885837

[157] Macaluso FS Orlando A Cottone M. Anti-interleukin-12 and anti-interleukin-23 agents in crohn's disease. Expert Opin Biol Ther. (2019) 19:89–98. 10.1080/14712598.2019.1561850 30571147

[158] Misselwitz B Juillerat P Sulz MC Siegmund B Brand S. Emerging treatment options in inflammatory bowel disease: janus kinases, stem cells, and more. Digestion. (2020) 1–14. 10.1159/000507782 32570252

[159] Burgdorf B Schlott S Ivanov IH Dissemond J. Successful treatment of a refractory pyoderma gangrenosum with risankizumab. Int Wound J. (2020) 17:1086–8. 10.1111/iwj.13359 32266771 PMC7948586

[160] John JM Sinclair RD. Tildrakizumab for treatment of refractory pyoderma gangrenosum of the penis and polymyalgia rheumatica: killing two birds with one stone. Australas J Dermatol. (2020) 61:170–1. 10.1111/ajd.13196 31729012

[161] Kok Y Nicolopoulos J Varigos G Howard A Dolianitis C. Tildrakizumab in the treatment of PASH syndrome: a potential novel therapeutic target. Australas J Dermatol. (2020) 61:e373–4. 10.1111/ajd.13285 32285437

[162] Kok Y Nicolopoulos J Howard A Varigos G Kern J Dolianitis C. Tildrakizumab in the treatment of moderate-to-severe hidradenitis suppurativa. Australas J Dermatol. (2020). 10.1111/ajd.13377 32627843

[163] Ismail FF Sinclair RD Pinczewski J. Refractory lupus erythematosus tumidus responsive to tildrakizumab. Dermatol Ther. (2019) 32:e13070. 10.1111/dth.13070 31442369

[164] Ismail FF Sinclair R. Clinical healing of erosive oral lichen planus with tildrakizumab implicates the interleukin-23/interleukin-17 pathway in the pathogenesis of lichen planus. Australas J Dermatol. (2020) 61:e244–5. 10.1111/ajd.13183 31651043

[165] Kerkemeyer KL Pinczewski J Sinclair R. Successful treatment of recalcitrant lichen planus pemphigoides with tildrakizumab. Australas J Dermatol. (2020) 61:e366–8. 10.1111/ajd.13263 32141608

[166] Jerjen R Moodley A Sinclair R. Repigmentation of acrofacial vitiligo with subcutaneous tildrakizumab. Australas J Dermatol. (2020) 61:e446–8. 10.1111/ajd.13346 32441048

[167] Ismail FF May J Moi J Sinclair R. Clinical improvement in psoriatic nail disease and psoriatic arthritis with tildrakizumab treatment. Dermatol Ther. (2020) 33:e13216. 10.1111/dth.13216 31899569

[168] Kerkemeyer KLS Sinclair R. Treatment of chronic alopecia areata with tildrakizumab: an open-label pilot study. Int J Dermatol. (2020) 59:e136–7. 10.1111/ijd.14826 32124974

[169] Trindade de Carvalho L Meah N Wall D Sinclair R. Recalcitrant lichen planopilaris and frontal fibrosing alopecia responding to tildrakizumab. Dermatol Ther. (2020) e13694. 10.1111/dth.13694 32458516

