# Supplementary material for: A Review of the Efficacy and Safety for Biologic Agents Targeting IL-23 in Treating Psoriasis With the Focus on Tildrakizumab
Source: Front Med (Lausanne). 2021 Aug 10;8:702776. doi: 10.3389/fmed.2021.702776 (PMC8383205; doi:10.3389/fmed.2021.702776)
Supplement: Supplementary file 1 [file Data_Sheet_1.docx]

**Appendix**. Summary of the PASI75, 90, and 100 responses, and achieving of PGA score 0/1 at weeks 12, 16, and/or 28 for biologic agents versus placebo in clinical trials evaluating the efficacy of IL-23, IL-23/12, IL-17 and TNF-α inhibitors

| **Reference** | **Phase** | **Biologic** | **Structure** | **Target** | **Control Arm; sample size (n=)** | **Treatment/Control Arm; sample size** | **Dosing Scheme** | **Baseline PASI** | **Week** | **PASI 75** | | | | | | **PASI 90** | | | | | | **PASI 100** | | | | | | **PGA Score more than 0 or 1** | | | | | |
| --- | --- | --- | --- | --- | --- | --- | --- | --- | --- | --- | --- | --- | --- | --- | --- | --- | --- | --- | --- | --- | --- | --- | --- | --- | --- | --- | --- | --- | --- | --- | --- | --- | --- |
|  |  |  |  |  |  |  |  |  |  | **Week 12** | | **Week 16** | | **Week 28** | | **Week 12** | | **Week 16** | | **Week 28** | | **Week 12** | | **Week 16** | | **Week 28** | | **Week 12** | | **Week 16** | | **Week 28** | |
|  |  |  |  |  |  |  |  |  |  | **Intervention (% n)** | **Placebo (% n)** | **Intervention (% n)** | **Placebo (% n)** | **Intervention (% n)** | **Placebo (% n)** | **Intervention (% n)** | **Placebo (% n)** | **Intervention (% n)** | **Placebo (% n)** | **Intervention (% n)** | **Placebo (% n)** | **Intervention (% n)** | **Placebo (% n)** | **Intervention (% n)** | **Placebo (% n)** | **Intervention (% n)** | **Placebo (% n)** | **Intervention (% n)** | **Placebo (% n)** | **Intervention (% n)** | **Placebo (% n)** | **Intervention (% n)** | **Placebo (% n)** |
| **Resurface 1 [**[**103**](#_ENREF_103)**]** | **3** | **Tildrakizumab** | **monoclonal IgG1 Kappa** | **IL-23p19** | **Placebo; n= 154** | **Tildrakizumab 100 mg; n=309** | **100 mg/ Weeks 0, 4** | **20** |  | **64** | **6** |  |  | **80 (N=299)** | **Placebo to tildrakizumab 100 mg (N=74) = 77**  **Placebo to tildrakizumab 200 mg (N=72)= 86** | **35** | **3** |  |  | **52 (N=299)** | **Placebo to tildrakizumab 100 mg (N=74) = 59**  **Placebo to tildrakizumab 200 mg (N=72)= 52** | **14** | **1** |  |  | **24 (N=299)** | **Placebo to tildrakizumab 100 mg (N=74) = 31**  **Placebo to tildrakizumab 200 mg (N=72)= 26** | **58** | **7** |  |  | **66** | **n/a** |
|  |  |  |  |  |  | **Tildrakizumab 200 mg; n=308** | **200 mg/ Weeks 0, 4** | **20.7** |  | **62** |  |  |  | **82 (N=298)** |  | **35** |  |  |  | **59 (N=298)** |  | **14** |  |  |  | **32 (N=298)** |  | **59** |  |  |  | **69** |  |
| **Resurface 2 [**[**103**](#_ENREF_103)**]** |  |  |  |  | **Placebo; n= 156** | **Tildrakizumab 100 mg; n=307** | **100 mg/ Weeks 0, 4** | **20.5** |  | **61** | **6** |  |  | **73 (N=294)** | **Placebo to tildrakizumab 100 mg (N=69)= 55**  **Placebo to tildrakizumab 200 mg (N=72)=69** | **39** | **1** |  |  | **56 (N=294)** | **Placebo to tildrakizumab 100 mg (N=69)= 39**  **Placebo to tildrakizumab 200 mg (N=72)= 49** | **12** | **0** |  |  | **23 (N=294)** | **Placebo to tildrakizumab 100 mg (N=69)= 14**  **Placebo to tildrakizumab 200 mg (N=72)= 19** | **55** | **4** |  |  | **65** | **n/a** |
|  |  |  |  |  |  | **Tildrakizumab 200 mg; n=314** | **200 mg/ Weeks 0, 4** | **19.8** |  | **66** |  |  |  | **73 (N=299)** |  | **37** |  |  |  | **58 (N=299)** |  | **12** |  |  |  | **27 (N=299)** |  | **59** |  |  |  | **69** |  |
|  |  |  |  |  |  | **Etanercept 50 mg; n=313** | **50 mg/ twice a week for 12 weeks; then 50 mg/ once a week for 16 weeks** | **20.2** |  | **48** |  |  |  | **54** |  | **21** |  |  |  | **31** |  | **5** |  |  |  | **11** |  | **48** |  |  |  | **45** |  |
| **Papp et al. (2015) [**[**102**](#_ENREF_102)**]** | **2b** |  |  |  | **Placebo; n=46** | **Tildrakizumab 5 mg; n=42** | **5 mg/ Weeks 0, 4, every 12 weeks thereafter** |  |  | **24** | **4** | **33** | **4** |  |  |  |  | **12** | **2** |  |  |  |  |  |  |  |  |  |  | **33** | **2** |  |  |
|  |  |  |  |  |  | **Tildrakizumab 25 mg; n=92** | **25 mg/ Weeks 0, 4, every 12 weeks thereafter** |  |  | **59** |  | **64** |  |  |  |  |  | **25** |  |  |  |  |  |  |  |  |  |  |  | **58** |  |  |  |
|  |  |  |  |  |  | **Tildrakizumab 100 mg; n=89** | **100 mg/ Weeks 0, 4, every 12 weeks thereafter** |  |  | **61** |  | **66** |  |  |  |  |  | **39** |  |  |  |  |  |  |  |  |  |  |  | **62** |  |  |  |
|  |  |  |  |  |  | **Tildrakizumab 200 mg; n=86** | **200 mg/ Weeks 0, 4, every 12 weeks thereafter** |  |  | **72** |  | **74** |  |  |  |  |  | **52** |  |  |  |  |  |  |  |  |  |  |  | **74** |  |  |  |
| **Kopp et al. (2015) [**[**98**](#_ENREF_98)**]** | **1** |  |  |  | **Placebo; n=20** | **Tildrakizumab 3 mg/kg; n=6** | **3 mg/kg - weeks 0,4 then every 4 weeks thereafter** |  | **16** | **100** |  |  |  |  |  | **83** |  |  |  |  |  |  |  |  |  |  |  |  |  |  |  |  |  |
|  |  |  |  |  |  | **Tildrakizumab 10 mg/kg; n=5** | **10 mg/kg - weeks 0,4 then every 4 weeks thereafter** |  | **16** | **60** |  |  |  |  |  | **20** |  |  |  |  |  |  |  |  |  |  |  |  |  |  |  |  |  |
| **Voyage 1 [**[**69**](#_ENREF_69)**]** | **3** | **Guselkumab** | **monoclonal IgG1 lambda** |  | **Placebo; n=174** | **Guselkumab 100 mg; n=329** | **100 mg/ Weeks 0, 4, every 8 weeks thereafter** | **22.1** |  |  |  | **91.2** | **5.7** |  |  |  |  | **73.3** | **2.9** |  |  |  |  | **37.4** | **0.6** |  |  |  |  | **85.1** | **6.9** |  |  |
| **Voyage 2 [**[**71**](#_ENREF_71)**]** | **3** |  |  |  | **Placebo; n=248** | **Guselkumab 100 mg; n=496** | **100 mg/ Weeks 0, 4, every 8 weeks thereafter** | **21.9** |  |  |  | **86.3** | **8.1** |  |  |  |  | **70** | **2.4** |  |  |  |  | **34.1** | **0.8** |  |  |  |  | **84.1** | **8.5** |  |  |
| **UltIMMa-1 [**[**76**](#_ENREF_76)**]** | **3** | **Risankizumab** | **monoclonal IgG1** |  | **Placebo; n=102** | **Risankizumab 150 mg; n=304** | **150 mg/ weeks 0,4 then every 12 weeks thereafter** | **20.6** |  | **86.8** | **9.8** |  |  |  |  |  |  | **75.3** | **4.9** |  |  |  |  | **35.9** | **0** |  |  | **82.2** | **8.8** | **87.8** | **7.8** |  |  |
| **UltIMMa-2 [**[**76**](#_ENREF_76)**]** | **3** |  |  |  | **Placebo; n=98** | **Risankizumab 150 mg; n=294** | **150 mg/ weeks 0,4 then every 12 weeks thereafter** | **20.5** |  | **88.8** | **8.2** |  |  |  |  |  |  | **74.8** | **2** |  |  |  |  | **50.7** | **2** |  |  | **82.3** | **9.2** | **83.7** | **5.1** |  |  |
| **Reich et al. (2019) [**[**79**](#_ENREF_79)**]** | **2** | **Mirikizumab** | **monoclonal IgG4-variant** |  | **Placebo; n=52** | **Mirikizumab 100 mg; n=51** | **100 mg/ week 0,8** | **20.3** |  |  |  | **78** | **4** |  |  |  |  | **59** | **0** |  |  |  |  | **31** | **0** |  |  |  |  | **71** | **2** |  |  |
|  |  |  |  |  |  | **Mirikizumab 300 mg; n=51** | **300 mg/ week 0,8** | **18.4** |  |  |  | **75** |  |  |  |  |  | **67** |  |  |  |  |  | **31** |  |  |  |  |  | **69** |  |  |  |
| **Phoenix-1 [**[**51**](#_ENREF_51)**]** | **3** | **Ustekinumab** | **human monoclonal IgG1 kappa** | **IL-23p40 and IL-12p40** | **Placebo; n=255** | **Ustekinumab 90 mg; n=256** | **90 mg/ week 0,4 and every 12 weeks thereafter** | **19.7** |  | **66.4** | **3.1** |  |  | **78.6** | **n/a** | **36.7** | **2** |  |  | **55.6** | **n/a** | **10.9** | **0** |  |  | **29.2** | **n/a** | **61.7** | **3.9** |  |  | **66.3** |  |
| **Phoenix-2[**[**52**](#_ENREF_52)**]** | **3** |  |  |  | **Placebo; n=410** | **Ustekinumab 90 mg; n=411** | **90 mg/ week 0,4 and every 12 weeks thereafter** | **20.1** |  | **75.7** | **3.7** |  |  | **78.5** | **n/a** | **50.9** | **0.7** |  |  | **54.3** | **n/a** | **18.2** | **0** |  |  | **29.5** | **n/a** | **73.5** | **4.9** |  |  | **70** | **n/a** |
| **FIXTURE [**[**134**](#_ENREF_134)**]** | **3** | **Secukinumab** | **recombinant human monoclonal IgG1 kappa** | **IL-17** | **Placebo; n=324** | **Secukinumab 150 mg; n=327** | **150 mg/ weeks 0-4 then every 4 weeks thereafter** | **23.7** |  | **67** | **4.9** |  |  |  |  | **41.9** | **1.5** |  |  |  |  | **14.4** | **0** |  |  |  |  | **51.1** | **2.8** |  |  |  |  |
|  |  |  |  |  |  | **Secukinumab 300 mg; n=323** | **300 mg/ weeks 0-4 then every 4 weeks thereafter** | **23.9** |  | **77.1** |  |  |  |  |  | **54.2** |  |  |  |  |  | **24.1** |  |  |  |  |  | **62.5** |  |  |  |  |  |
|  |  |  |  |  |  | **Etanercept 50 mg; n=323** | **50 mg/ weeks 0-4 then every 4 weeks thereafter** | **23.2** |  | **44** |  |  |  |  |  | **20.7** |  |  |  |  |  | **4.3** |  |  |  |  |  | **27.2** |  |  |  |  |  |
| **UNCOVER-1 [**[**122**](#_ENREF_122)**]** | **3** | **Ixekizumab** | **humanized IgG subclass 4** |  | **placebo; n=431** | **Ixekizumab; n=433** | **160 mg x 1, 80 mg (q2w** | **20** |  | **89.1** | **3.9** |  |  |  |  | **70.9** | **0.5** |  |  |  |  | **35.3** | **0** |  |  |  |  | **81.8** | **3.2** |  |  |  |  |
|  |  |  |  |  |  | **Ixekizumab; n=432** | **160 mg x 1, 80 mg (q4w** | **20** |  | **82.6** |  |  |  |  |  | **64.6** |  |  |  |  |  | **33.6** |  |  |  |  |  | **76.4** |  |  |  |  |  |
| **AMAGINE-1 [**[**135**](#_ENREF_135)**]** | **3** | **Brodalumab** | **human monoclonal IgG subclass 2 kappa** |  | **placebo; n=220** | **Brodalumab 140 mg; n=219** | **140 mg/ q2w** | **20** |  | **60.3** | **2.7** |  |  |  |  | **42.5** | **0.9** |  |  |  |  | **23.3** | **0.5** |  |  |  |  | **53.9** | **1.4** |  |  |  |  |
|  |  |  |  |  |  | **Brodalumab 210 mg; n=222** | **210 mg/ q2w** | **19.4** |  | **83.3** |  |  |  |  |  | **70.3** |  |  |  |  |  | **41.9** |  |  |  |  |  | **75.7** |  |  |  |  |  |
| **BE ABLE 1 [**[**136**](#_ENREF_136)**]** | **2b** | **Bimekizumab** | **humanized monoclonal IgG1** |  | **Placebo; n=42** | **Bimekizumab 160 mg; n=40** | **160 mg/ q4w** | **20.6** |  | **81.4** | **4.8** |  |  |  |  | **67.4** | **0** |  |  |  |  | **27.9** | **0** |  |  |  |  | **74.4** | **4.8** |  |  |  |  |
|  |  |  |  |  |  | **Bimekizumab 320 mg; n=43** | **320 mg/ q4w** | **19.4** |  | **93.1** |  |  |  |  |  | **79.1** |  |  |  |  |  | **55.8** |  |  |  |  |  | **86** |  |  |  |  |  |
| **FIXTURE [**[**134**](#_ENREF_134)**]** | **3** | **Etanercept** | **dimeric fusion protein consisting of the extracellular ligand-binding portion of the human 75 kilodalton (p75) tumor necrosis factor receptor (TNFR) linked to the Fc portion of human IgG1** | **TNF-alpha** | **Placebo; n=324** | **Etanercept 50 mg; n=323** | **50 mg/ weeks 0-4 then every 4 weeks thereafter** | **23.2** |  | **44** | **4.9** |  |  |  |  | **20.7** | **1.5** |  |  |  |  | **4.3** | **0** |  |  |  |  | **27.2** | **2.8** |  |  |  |  |
| **EXPRESS II [**[**72**](#_ENREF_72)**]** | **3** | **Infliximab** | **chimeric IgG1 kappa monoclonal antibody (composed of human constant and murine variable regions)** |  | **Placebo; n=208** | **Infliximab 5 mg/kg; n=314** | **5 mg/kg; weeks 0,2,6 then every 8 weeks thereafter** | **20.4** | **10** | **75.5** | **1.9** |  |  |  |  | **45.2** | **0.5** |  |  |  |  |  |  |  |  |  |  |  |  |  |  |  |  |
| **REVEAL[**[**138**](#_ENREF_138)**]** | **3** | **Adalimumab** | **recombinant human monoclonal IgG1; IgG1:Kappa constant regions** |  | **Placebo; n=398** | **Adalimumab 40 mg; n=814** | **40 mg q2w** | **19** | **12** |  |  |  |  |  |  | **37** | **2** |  |  |  |  | **14** | **<1** |  |  |  |  | **60** | **4** |  |  |  |  |
| **CIMPACT[**[**139**](#_ENREF_139)**]** | **3** | **Certolizumab pegol** | **recombinant, humanized antibody Fab' fragment** |  | **Placebo; n= 57** | **Certolizumab pegol 200 mg; n=165** | **200 mg q2w** | **21.4** | **12, 16** | **61.3** | **5** | **68.2** | **3.8** |  |  | **31.2** | **0.2** | **39.8** | **0.3** |  |  |  |  |  |  |  |  | **39.8** | **1.9** | **48.3** | **3.4** |  |  |
| **GO-VIBRANT [**[**140**](#_ENREF_140)**]** | **3** | **Golimumab** | **human monoclonal IgG1 kappa** |  | **Placebo; n=239** | **Golimumab 2 mg/kg; n=241** | **2 mg/kg, weeks 0,4 then every 8 weeks thereafter** |  | **14** | **59.2** | **13.6** |  |  |  |  | **39.3** | **6.6** |  |  |  |  | **16.8** | **4.5** |  |  |  |  |  |  |  |  |  |  |
